# Toxicity profiles of antibody-drug conjugates for anticancer treatment: a systematic review and meta-analysis

**DOI:** 10.1093/jncics/pkad069

**Published:** 2023-09-26

**Authors:** Yukio Suzuki, Susu Zhou, Yukihide Ota, Matthew Harrington, Etsuko Miyagi, Hisato Takagi, Toshiki Kuno, Jason D Wright

**Affiliations:** Division of Gynecologic Oncology, Department of Obstetrics and Gynecology, Columbia University College of Physicians and Surgeons, New York, NY, USA; Department of Obstetrics and Gynecology, Yokohama City University Graduate School of Medicine, Yokohama, Japan; Department of Medicine, Icahn School of Medicine at Mount Sinai, Mount Sinai Beth Israel, New York, NY, USA; Department of Obstetrics and Gynecology, Yokohama City University Graduate School of Medicine, Yokohama, Japan; Department of Obstetrics and Gynecology, Washington University School of Medicine, St Louis, MO, USA; Department of Medicine, Icahn School of Medicine at Mount Sinai, Mount Sinai Beth Israel, New York, NY, USA; Department of Obstetrics and Gynecology, Yokohama City University Graduate School of Medicine, Yokohama, Japan; Department of Cardiovascular Surgery, Shizuoka Medical Center, Shizuoka, Japan; Division of Cardiology, Montefiore Medical Center, Albert Einstein College of Medicine, New York, NY, USA; Division of Gynecologic Oncology, Department of Obstetrics and Gynecology, Columbia University College of Physicians and Surgeons, New York, NY, USA

## Abstract

**Background:**

Antibody-drug conjugates are attractive targeted agents in anticancer treatment because of their unique mechanism of action and reduced toxicity. Little is known about the spectrum of adverse events associated with antibody-drug conjugates, despite tens of clinical trials.

**Methods:**

A systematic review of randomized controlled trials evaluating antibody-drug conjugate efficacy in anticancer treatment was conducted. PubMed, EMBASE, and ClinicalTrial.gov were searched for relevant studies. Meta-analyses assessed the odds ratios (ORs) of 12 treatment-related symptoms and toxicities in patients treated with antibody-drug conjugates compared with those receiving other anticancer agents without antibody-drug conjugates. All-grade and high-grade (grade ≥3) toxicities were examined.

**Results:**

Twenty studies involving 10 075 patients were included. Compared with control groups, antibody-drug conjugates were associated with a higher risk of all-grade fatigue (OR = 1.25, 95% confidence interval [CI] = 1.08 to 1.45), anorexia (OR = 1.36, 95% CI = 1.09 to 1.69), nausea (OR = 1.46, 95% CI = 1.09 to 1.97), and sensory neuropathy (OR = 2.18, 95% CI = 1.27 to 3.76) as treatment-related symptoms. Patients treated with antibody-drug conjugates had a statistically significantly lower risk of all-grade febrile neutropenia (OR = 0.46, 95% CI = 0.22 to 0.96). Conversely, they had a higher risk of thrombocytopenia (OR = 2.07, 95% CI = 1.00 to 4.31), increased alanine aminotransferase (OR = 2.51, 95% CI = 1.84 to 3.40), and increased aspartate aminotransferase (OR = 2.83, 95% CI = 2.04 to 3.93). Subgroup analysis showed a similar toxicity profile when comparing the solid tumors with hematologic malignancy groups and the antibody-drug conjugate vs antibody-drug conjugate plus chemotherapy groups, except for some neurologic and hematologic adverse events.

**Conclusions:**

This comprehensive profile of adverse events associated with antibody-drug conjugate–based treatment shows an increase in various types of all-grade treatment-related symptoms and adverse events, although no increase in high-grade adverse events was seen.

The use of antibody-drug conjugates in cancer treatment has seen a substantial rise, yet the toxicity profiles of these agents remain largely unknown. Antibody-drug conjugates constitute a class of anticancer agents that consist of cytotoxic payloads, monoclonal antibodies, and chemical linkers joining them. Antibody-drug conjugates are directed toward a target antigen overexpressed on the cancer cell surface (1,2). When antibody-drug conjugates are delivered directly to the cancer cell, they are internalized and release payloads to destroy the target cells, which reduces off-target toxicities in patients by minimizing their systemic exposure (3,4). Owing to their superior tumor-to-normal tissue selectivity and efficacy over conventional cancer chemotherapeutics, antibody-drug conjugate–based therapies are becoming a transformative approach to cancer treatment (2). The pace of antibody-drug conjugate development is accelerating, with 14 different antibody-drug conjugates currently approved by the US Food and Drug Administration for various treatment settings in both hematologic and solid tumors and hundreds of studies and clinical trials currently underway (5,6).

Despite the growing interest in and enthusiasm for antibody-drug conjugates, challenges remain to find strategies for further optimization of molecular targeted therapies with greater efficacy and less toxicity. Although antibody-drug conjugates may reduce systemic toxicities compared with conventional cytotoxic drugs by minimizing off-target effects, previous studies have shown that antibody-drug conjugates are associated with unexpected dose-limiting toxicities (2). These toxicities include hematologic, hepatic, kidney, gastrointestinal, neurologic, and ophthalmic events, which are largely attributed to the premature release of cytotoxic payloads into the circulation because of the instability of the cleavable linkers (7). In addition, antibody-drug conjugates may be associated with a risk of immunogenicity, and immune responses induced by their components (antibodies or cytotoxic agents) may cause secondary damage (8). Although most adverse events are mild to moderate, some may require dose reduction or discontinuation of treatment. Further, even a treatment-related death may occur once severe adverse events begin to occur (9,10). With the increasing use of antibody-drug conjugates in cancer treatment, it is imperative for clinicians to have a comprehensive knowledge of the toxicity associated with antibody-drug conjugate–based regimens.

We conducted a systematic review and meta-analysis of treatment-related adverse events in patients receiving antibody-drug conjugate–based therapy to provide oncologists with a better understanding of how to monitor and manage these antibody-drug conjugate–related toxicities.

## Methods

This meta-analysis was conducted under using the Preferred Reporting Items for Systematic Reviews and Meta-Analyses (11) reporting guidelines and registered in the International Prospective Register of Systematic Reviews under registration No. CRD42023397264. This study used deidentified public data and was classified as nonhuman-subjects research.

### Data source and search strategy

We used a 2-level strategy to search for all randomized controlled trials investigating the effectiveness of antibody-drug conjugates. As a first step, we used PubMed, EMBASE, and ClinicalTrial.gov for a comprehensive search on January 10, 2023. We used (“antibody-drug conjugate” or “ADC”) and (“cancer” or “malignancy”) as key search terms. We then performed an additional manual search of secondary sources, including studies referenced in articles retrieved during the first step, to accomplish a comprehensive review.

### Study selection

Studies meeting the following criteria were eligible for inclusion in our review: (1) the study was published in a peer-reviewed journal, (2) the study was a randomized controlled trial in which participants were randomly assigned to experimental (with antibody-drug conjugate) and control (without antibody-drug conjugate) groups, (3) the study was registered in ClinicalTrials.gov, (4) the study enrolled patients treated with antibody-drug conjugates in at least 1 experimental arm, and (5) the study had comprehensive profiles of adverse events as a final report in ClinicalTrials.gov. Full text written in English was retained for the analysis.

### Data extraction

Data were independently extracted from eligible studies by 2 investigators (S.Z. and Y.S.). Any discrepancies between reviewers were resolved by consensus. The following information was recorded for each study: first author’s name, publication year, trial phase, blinding, malignancy type, number of patients available for analysis, type of the antibody-drug conjugate in the treatment arm, placebo or treatment drug in control arm, and the grade of toxicity. The Cochrane Risk of Bias Tool was used to evaluate the risk of bias for each randomized controlled trial (12). The quality of the included trials was independently assessed by 2 investigators (S.Z. and Y.S.).

### Statistical analysis

The primary outcome of this meta-analysis was the pooled odds ratio (OR) of each all-grade adverse event associated with the use of antibody-drug conjugates. Additionally, we examined the pooled odds ratios of high-grade adverse events (grade ≥3). The number of treated patients and the number of patients who developed adverse events in each treatment arm were recorded from each trial. Adverse events reported in at least 3 randomized controlled trials were selected as toxicity endpoints for meta-analysis to ensure the minimum generalizability of the pooled odds ratio. The odds ratio and corresponding 95% confidence interval (CI) were calculated using random-effects models. *P* < .05 was considered statistically significant. Cochran *Q* test and *I*^2^ statistics were used to assess the heterogeneity in each adverse event analysis. *I*^2^ values of greater than 50% were considered evidence of substantial heterogeneity in the study. Egger linear regression tests and funnel plots were applied in ProMeta, version 3.0, to adverse events to show statistically significant differences in risk and evaluate publication bias. Subgroup analyses were also conducted based on cancer type (solid tumor vs hematologic malignancy) and antibody-drug conjugate regimen (antibody-drug conjugate vs antibody-drug conjugate plus chemotherapy). The *P* value for interaction was calculated to determine whether the risk of each adverse event differed between the subgroups. *P < *.1 for interaction was considered statistically significant in our study (13). We used RevMan, version 5.4 (The Cochrane Collaboration, Copenhagen, Denmark) for these analyses (14).

## Results

### Eligible studies and patient characteristics

**Figure 1.**
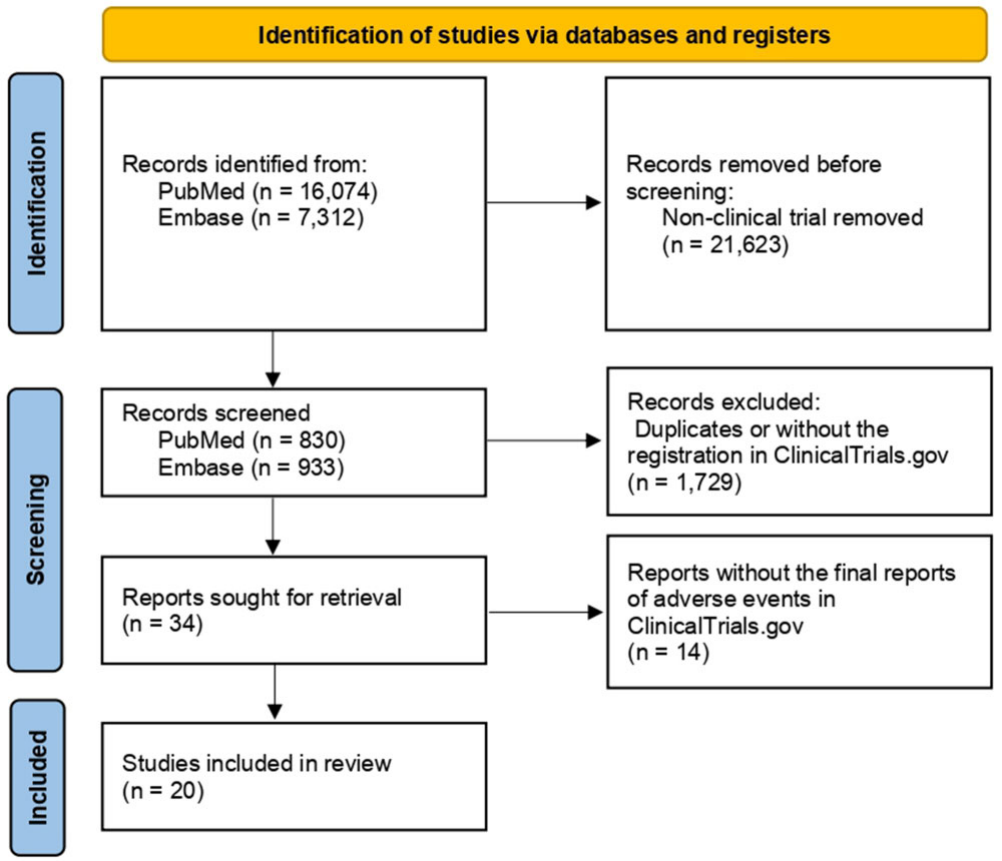
Flow diagram of study selection

The systematic literature search identified 23 386 records, from which 34 potentially eligible studies were collected after intensive screening. The duplicate citations and papers without full text were removed. Only studies registered in ClinicalTrials.gov were included to be able to analyze adverse events comprehensively. Ultimately, 20 randomized controlled trials comprising 10 075 patients met the predefined criteria were included in this meta-analysis. We included 5745 patients who had received any antibody-drug conjugate (experimental arm) and 4330 patients who did not (control arm) (15-34). Details of the retrieval process are shown in Figure 1. The risk of bias of each study was assessed and is summarized in Supplementary Figure 1 (available online). The Egger test and funnel plot showed evidence of publication bias in febrile neutropenia (*P* = .030) and neutropenia (*P* = .048).

The baseline characteristics of the included studies are presented in Table 1. Of 20 clinical trials included in this study, 14 were phase 3 and 6 were phase 2 trials, 16 were open label, and 4 were double-blind studies. Fourteen studies (70.0%) enrolled patients with solid tumors, and 6 (30.0%) enrolled patients with hematologic malignancies. In terms of malignancy types, the studies included patients with breast cancer (n = 6), lymphoma (n = 5), small cell lung cancer (n = 3), acute lymphoblastic leukemia (n = 1), gastric cancer (n = 1), glioblastoma (n = 1), mesothelioma (n = 1), renal cell carcinoma (n = 1), and ovarian cancer (n = 1). Of the included studies, antibody-drug conjugate monotherapy was used in 15 (75.0%) experimental arms and combination therapy of an antibody-drug conjugate with any anticancer agent was used in 7 (35.0%) arms. Regarding the control arms, 2 (10.0%) studies used placebo and another 18 (90.0%) studies used an active comparator arm in which patients were given a corresponding unconjugated antibody, hormone therapy, or cytotoxic or targeted therapy. Various types of antibody-drug conjugates were used: trastuzumab emtansine (n = 5), brentuximab vedotin (n = 4), rovalpituzumab tesirine (n = 2), inotuzumab ozogamicin (n = 2), trastuzumab deruxtecan (n = 1), depatuxizumab mafodotin (n = 1), anetumab ravtansine (n = 1), AGS-16C3F (n = 1), IMGN901 (n = 1), mirvetuximab soravtansine (n = 1), and sacituzumab govitecan (n = 1).

**Table 1.** Main characteristics of the included randomized control trials

Author and y	Phase	Blinding	NCT No.	Cancer type	Mean (SD) age, y		Female, %		Treatment setting		Patients affected,^a^ No. (%)					
					Antibody-drug conjugate	Control	Antibody-drug conjugate	Control	Antibody-drug conjugate (payload)	Control	Antibody-drug conjugate			Control		
											At risk	All grade	High grade	At risk	All grade	High grade
Hurvitz, 2013 (15)	2	Open label	00679341	Breast	54.3 (12.6)	52.1 (10.7)	100	100	Trastuzumab emtansine (DM1)	Trastuzumab + docetaxel	69	66 (95.65)	14 (20.29)	66	66 (100.00)	17 (25.76)
Moskowitz, 2015 (16)	3	Double-blind	01100502	Hodgkin lymphoma	33 (18 to 71)^b^	32 (18 to 76)^b^	53.9	40.9	Brentuximab vedotin (MMAE)	Placebo	167	151 (90.42)	43 (25.75)	160	127 (79.38)	21 (13.13)
Krop, 2017 (17)	3	Open label	01419197	Breast	53.3 (10.4)	54.3 (10.8)	99.3	99.5	Trastuzumab emtansine (DM1)	Tamoxifen or aromatase inhibitor or aromatase inhibitor + LHRH agonist	497	445 (89.54)	121 (24.35)	184	148 (80.43)	41 (22.28)
Dieras, 2017 (18)	3	Open label	00829166	Breast	52.2 (11.0)	53.2 (10.8)	99.8	99.2	Trastuzumab emtansine (DM1)	Lapatinib + capecitabine	490	474 (96.73)	92 (18.78)	488	471 (96.52)	99 (20.29)
Perez, 2017 (19)	3	Double-blind	01120184	Breast	52.6 (11.4)	54.2 (11.3)	99.4	99.2	Trastuzumab emtansine (DM1) + placebo or pertuzumab	Trastuzumab + taxane	727	704 (96.84)	179 (24.62)	353	342 (96.88)	81 (22.95)
Prince, 2017 (20)	3	Open label	01578499	T-cell lymphoma	59.4 (13.8)	56.6 (14.43)	50.0	45.2	Brentuximab vedotin (MMAE)	Methotrexate or bexarotene	66	60 (90.91)	18 (27.27)	62	51 (82.26)	18 (29.03)
Socinski, 2017 (21)	2	Open label	01237678	Small cell lung cancer	NA	NA	42.6	46.8	IMGN901 (DM1) + carboplatin + etoposide	Carboplatin + etoposide	94	91 (96.81)	54 (57.45)	47	43 (91.49)	23 (48.94)
Dang, 2018 (22)	3	Open label	01232556	Non-Hodgkin lymphoma	68.6 (12.29)	66.9 (11.40)	45.2	43.6	Inotuzumab ozogamicin (calicheamicin) + rituximab	Rituximab + gemcitabine or rituximab + bendamustine	164	155 (94.51)	61 (37.2)	167	158 (94.61)	63 (37.72)
Horwitz, 2019 (23)	3	Double-blind	01777152	Lymphoma	NA	NA	41.2	33.2	Brentuximab vedotin (MMAE) + cyclophosphamide + doxorubicin + prednisone	Cyclophosphamide, doxorubicin, vincristine, and prednisone doxorubicin	223	220 (98.65)	89 (39.91)	226	218 (96.46)	90 (39.82)
Kantarjian, 2019 (24)	3	Open label	01564784	Acute lymphoblastic leukemia	45.9 (17.07)	45.6 (16.32)	44.5	35.7	Inotuzumab ozogamicin (calicheamicin)	Fludarabine, cytarabine, G-CSF, mitoxantrone + cytarabin or high-dose cytarabine	164	159 (96.95)	85 (51.83)	143	143 (100.00)	72 (50.35)
von Minckwitz, 2019 (25)	3	Open label	01772472	Breast	NA	NA	99.7	99.6	Trastuzumab emtansine (DM1)	Trastuzumab	740	719 (97.16)	94 (12.70)	720	634 (88.06)	58 (8.06)
Shitara, 2020 (26)	2	Open label	03329690	Gastric	64.2 (10.36)	64.9 (10.54)	24.0	24.2	Trastuzumab deruxtecan (DXd)	Irinotecan or paclitaxel	125	125 (100.00)	58 (46.40)	62	61 (98.39)	16 (25.81)
van den Bent, 2020 (27)	2	Open label	02343406	Glioblastoma	57.9 (8.15)	55.9 (11.04)	37.8	34.6	Depatuxizumab mafodotin (MMAF) with or without temozolomide	Temozolomide	172	160 (93.02)	69 (40.12)	21	20 (95.24)	5 (23.81)
Bardia, 2021 (28)	3	Open label	02574455	Breast	54.0 (11.34)	54.0 (11.69)	99.3	100	Sacituzumab govitecan (SN-38)	Eribulin, capecitabine, gemcitabine, or vinorelbine	258	256 (99.22)	69 (26.74)	224	213 (95.09)	64 (28.57)
Blackhall, 2021 (29)	3	Open label	03061812	Small cell lung cancer	63.0 (8.57)	63.4 (8.72)	35.5	41.9	Rovalpituzumab tesirine (tesirine)	Topotecan	287	245 (85.37)	160 (55.75)	129	118 (91.47)	74 (57.36)
Johnson, 2021 (30)	3	Double-blind	03033511	Small cell lung cancer	64.1 (8.40)	63.8 (8.20)	30.6	36.4	Rovalpituzumab tesirine (tesirine) + dexamethasone	Placebo	368	315 (85.60)	157 (42.66)	373	248 (66.49)	87 (23.32)
Kollmannsberger, 2021 (31)	2	Open label	02639182	Renal cell carcinoma	62.3 (9.1)	61.1 (8.9)	26.9	25.8	AGS-16C3F (MMAF)	Axitinib	66	65 (98.48)	26 (39.39)	65	64 (98.46)	31 (47.69)
Moore, 2021 (32)	3	Open label	02631876	Ovarian	62.7 (10.29)	62.9 (10.51)	100	100	Mirvetuximab soravtansine (DM4)	Paclitaxel or topotecan or doxorubicin	243	242 (99.59)	67 (27.57)	109	106 (97.25)	31 (28.44)
Ansell, 2022 (38)	3	Open label	01712490	Hodgkin lymphoma	38.8 (15.83)	40.2 (16.05)	43.1	40.6	Brentuximab vedotin (MMAE) + doxorubicin + vinblastine +dacarbazine	Doxorubicin, bleomycin, vinblastine, and dacarbazine	662	644 (97.28)	284 (42.90)	659	632 (95.90)	178 (27.01)
Kindler, 2022 (39)	2	Open label	02610140	Mesothelioma	66.1 (8.1)	65.6 (8.8)	26.5	24.4	Anetumab ravtansine (DM4)	Vinorelbine	163	161 (98.77)	56 (34.36)	72	68 (94.44)	25 (34.72)

a Per-protocol analysis population was used for the number of patients. DM1 = maitansine; DM4 = a maytansinoid; DXd = a topoisomerase I inhibitor; LHRH = luteinizing hormone-releasing hormone; MMAE = monomethyl auristatin E; MMAF = monomethyl auristatin F; NA = not applicable; SN-38 = an active metabolite of irinotecan.

b Age range.

### Clinically relevant treatment-related symptoms

**Figure 2.**
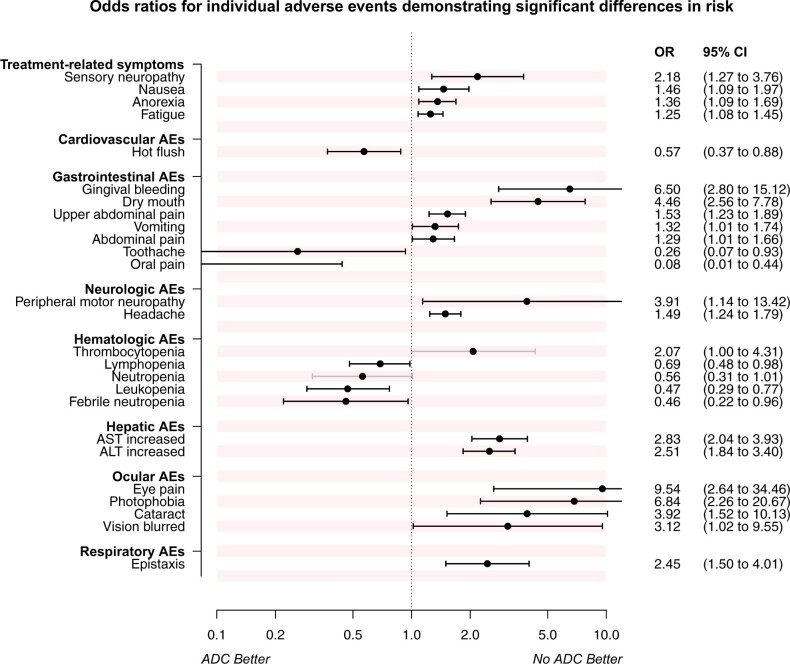
Forest plot of ORs for individual adverse events (AEs), demonstrating statistically significant differences in risk between patients treated with an antibody-drug conjugate (ADC) and those not treated with an antibody-drug conjugate. ALT = alanine aminotransferase; AST = aspartate aminotransferase; CI = confidence interval; OR = odds ratio.

First, we analyzed a core set of 12 clinically relevant symptoms recommended by the US National Cancer Institute for cross-study comparisons of symptomatic effects (35). Of these symptoms, fatigue, insomnia, anorexia (decreased appetite), nausea, constipation, and diarrhea were reported in all 20 randomized controlled trials; and dyspnea was reported in 19 randomized controlled trials. Data for pain were available in 14 randomized controlled trials, and data for sensory neuropathy and depression were available in 13 and 12 randomized controlled trials, respectively. Anxiety and cognitive problems were reported in 11 and 4 trials, respectively. Compared with control groups, antibody-drug conjugates were associated with a statistically significantly higher risk for all-grade fatigue (35.3% in antibody-drug conjugate arms vs 30.5% in control arms; OR = 1.25, 95% CI = 1.08 to 1.45), anorexia (18.1% in antibody-drug conjugate arms vs 13.7% in control arms; OR = 1.36, 95% CI = 1.09 to 1.69), nausea (41.7% in antibody-drug conjugate arms vs 33.3% in control arms; OR = 1.46, 95% CI = 1.09 to 1.97), and sensory neuropathy (21.3% in antibody-drug conjugate arms vs 13.8% in control arms; OR = 2.18, 95% CI = 1.27 to 3.76), whereas the risk of high-grade adverse events for each of these 4 symptoms was not statistically significantly increased (Figure 2;Supplementary Figure 2, available online). No statistically significant differences were found between groups for all-grade or high-grade insomnia, pain, dyspnea, cognitive disorder, anxiety, depression, constipation, or diarrhea (Table 2).

**Table 2.** Odds ratios of individual adverse events

	All-grade adverse events			High-grade adverse events		
	Studies, No.	OR (95% CI)	***P*** ^a^	Studies, No.	OR (95% CI)	***P*** ^a^
**Treatment-related symptoms**						
Fatigue	20	1.25 (1.08 to 1.45)	**.002**	13	1.04 (0.47 to 2.29)	.92
Insomnia	20	1.21 (0.97 to 1.51)	.10	NA	—	—
Pain	14	1.21 (0.89 to 1.64)	.23	9	1.34 (0.50 to 3.58)	.56
Anorexia	20	1.36 (1.09 to 1.69)	**.006**	9	1.31 (0.56 to 3.05)	.54
Dyspnea	19	0.96 (0.76 to 1.21)	.75	15	0.84 (0.46 to 1.53)	.57
Cognitive problems	4	0.38 (0.08 to 1.88)	.23	4	0.38 (0.08 to 1.88)	.23
Anxiety	11	0.94 (0.68 to 1.29)	.69	3	0.19 (0.03 to 1.20)	.08
Nausea	20	1.46 (1.09 to 1.97)	**.01**	14	1.30 (0.68 to 2.47)	.43
Depression	12	1.08 (0.87 to 1.36)	.48	3	1.24 (0.20 to 7.65)	.82
Sensory neuropathy	13	2.18 (1.27 to 3.76)	**.005**	6	3.17 (0.92 to 10.93)	.07
Constipation	20	1.13 (0.87 to 1.46)	.36	10	1.51 (0.76 to 2.99)	.24
Diarrhea	20	0.99 (0.56 to 1.74)	.97	18	0.78 (0.39 to 1.59)	.50
**Cardiovascular adverse events**						
Acute myocardial infarction	4	0.23 (0.05 to 1.13)	.07	4	0.23 (0.05 to 1.13)	.07
Angina pectoris	3	1.43 (0.23 to 9.14)	.70	3	1.43 (0.23 to 9.14)	.70
Atrial fibrillation	12	1.18 (0.52 to 2.68)	.68	12	1.22 (0.52 to 2.86)	.65
Cardiac arrest	7	1.19 (0.38 to 3.71)	.76	7	1.19 (0.38 to 3.71)	.76
Congestive heart failure	4	1.97 (0.40 to 9.79)	.41	4	1.97 (0.40 to 9.79)	.41
Deep vein thrombosis	10	1.28 (0.57 to 2.84)	.55	8	1.22 (0.52 to 2.87)	.65
Embolism	7	0.76 (0.26 to 2.25)	.62	7	0.76 (0.26 to 2.25)	.62
Flushing	3	1.15 (0.30 to 4.41)	.84	NA	—	—
Heart failure	5	1.52 (0.45 to 5.18)	.50	5	1.52 (0.45 to 5.18)	.50
Hematoma	6	1.42 (0.41 to 4.96)	.58	6	1.42 (0.41 to 4.96)	.58
Hot flush	6	0.57 (0.37 to 0.88)	**.01**	NA	—	—
Hypertension	14	1.25 (0.81 to 1.92)	.31	4	0.68 (0.15 to 2.98)	.61
Hypotension	17	0.99 (0.66 to 1.51)	.98	15	1.07 (0.49 to 2.31)	.87
Lymphedema	6	0.55 (0.28 to 1.11)	.10	2	—	—
Myocardial infarction	6	1.03 (0.31 to 3.47)	.96	6	1.03 (0.31 to 3.47)	.96
Orthostatic hypotension	3	0.55 (0.09 to 3.47)	.52	3	0.55 (0.09 to 3.47)	.52
Palpitations	3	1.21 (0.40 to 3.61)	.74	NA	—	—
Pericardial effusion	9	1.35 (0.40 to 4.59)	.63	9	0.76 (0.30 to 1.92)	.56
Pericarditis	4	1.24 (0.27 to 5.77)	.79	4	1.24 (0.27 to 5.77)	.79
Shock hemorrhagic	3	2.20 (0.35 to 14.02)	.40	3	2.20 (0.35 to 14.02)	.40
Sinus tachycardia	9	1.52 (0.71 to 3.25)	.28	6	0.82 (0.23 to 2.92)	.76
Superior vena cava syndrome	4	0.97 (0.29 to 3.26)	.96	4	0.97 (0.29 to 3.26)	.96
Supraventricular tachycardia	5	1.87 (0.51 to 6.85)	.34	5	1.87 (0.51 to 6.85)	.34
Tachycardia	6	0.73 (0.32 to 1.63)	.44	3	1.28 (0.28 to 5.74)	.75
Unstable angina	3	0.53 (0.08 to 3.39)	.50	3	0.53 (0.08 to 3.39)	.50
**Gastrointestinal adverse events**						
Abdominal distension	7	1.14 (0.64 to 2.03)	.66	1	—	—
Abdominal pain	19	1.29 (1.01 to 1.66)	**.04**	19	1.38 (0.87 to 2.20)	.18
Abdominal pain, lower	5	1.77 (0.64 to 4.85)	.27	3	2.20 (0.35 to 14.02)	.40
Abdominal pain, upper	16	1.53 (1.23 to 1.89)	**.0001**	8	1.11 (0.36 to 3.46)	.85
Ascites	7	1.22 (0.59 to 2.52)	.60	5	1.55 (0.39 to 6.12)	.53
Colitis	9	2.16 (0.76 to 6.17)	.15	9	2.16 (0.76 to 6.17)	.15
Dry mouth	8	4.46 (2.56 to 7.78)	**<.00001**	NA	—	—
Dyspepsia	15	1.15 (0.85 to 1.56)	.38	1	—	—
Dysphagia	6	0.48 (0.18 to 1.25)	.13	3	1.04 (0.19 to 5.78)	.97
Enteritis	3	0.66 (0.10 to 4.22)	.66	3	0.66 (0.10 to 4.22)	.66
Gastric hemorrhage	4	1.01 (0.21 to 4.89)	.99	4	0.78 (0.16 to 3.87)	.76
Gastric ulcer	3	1.14 (0.18 to 7.24)	.89	3	1.14 (0.18 to 7.24)	.89
Gastroesophageal reflux disease	6	1.06 (0.61 to 1.82)	.84	NA	—	—
Gastrointestinal hemorrhage	8	1.38 (0.46 to 4.13)	.57	8	1.38 (0.46 to 4.13)	.57
Gastritis	6	1.35 (0.44 to 4.11)	.60	6	1.35 (0.44 to 4.11)	.60
Gingival bleeding	5	6.50 (2.80 to 15.12)	**<.0001**	NA	—	—
Hemorrhoids	5	0.98 (0.44 to 2.17)	.95	2	—	—
Ileus	5	1.16 (0.33 to 4.10)	.82	5	1.16 (0.33 to 4.10)	.82
Inguinal hernia	3	0.87 (0.14 to 5.56)	.88	2	—	—
Intestinal obstruction	7	0.84 (0.34 to 2.12)	.72	7	0.84 (0.34 to 2.12)	.72
Intestinal perforation	3	2.31 (0.36 to 14.78)	.38	3	2.31 (0.36 to 14.78)	.38
Large intestine obstruction	3	0.80 (0.20 to 3.14)	.75	3	0.80 (0.20 to 3.14)	.75
Large intestine perforation	3	0.69 (0.11 to 4.39)	.69	3	0.69 (0.11 to 4.39)	.69
Lower gastrointestinal hemorrhage	3	1.09 (0.17 to 6.93)	.93	3	1.09 (0.17 to 6.93)	.93
Oral pain	3	0.08 (0.01 to 0.44)	**.004**	NA	—	—
Small intestine obstruction	6	0.75 (0.30 to 1.85)	.53	6	0.73 (0.27 to 1.93)	.52
Stomatitis	17	0.69 (0.43 to 1.09)	.11	6	0.73 (0.23 to 2.33)	.60
Toothache	3	0.26 (0.07 to 0.93)	**.04**	NA	—	—
Upper gastrointestinal hemorrhage	7	1.82 (0.56 to 5.89)	.32	7	1.82 (0.56 to 5.89)	.32
Vomiting	20	1.32 (1.01 to 1.74)	**.04**	16	1.06 (0.64 to 1.74)	.83
**Neurologic adverse events**						
Ataxia	3	0.64 (0.11 to 3.62)	.61	2	—	—
Balance disorder	3	1.64 (0.29 to 9.44)	.58	1	—	—
Brain edema	4	0.84 (0.17 to 4.07)	.84	3	1.41 (0.23 to 8.62)	.71
Cerebrovascular accident	6	0.57 (0.16 to 1.98)	.38	6	0.57 (0.16 to 1.98)	.38
Depressed level of consciousness	5	0.57 (0.14 to 2.36)	.44	4	0.63 (0.13 to 3.07)	.57
Dizziness	18	1.18 (0.97 to 1.42)	.09	8	1.58 (0.57 to 4.41)	.38
Dysarthria	3	0.82 (0.14 to 4.96)	.83	1	—	—
Dysgeusia	15	1.17 (0.75 to 1.81)	.49	NA	—	—
Epilepsy	5	1.14 (0.31 to 4.16)	.85	5	1.14 (0.31 to 4.16)	.85
Facial paralysis	3	0.50 (0.08 to 3.20)	.47	3	0.50 (0.08 to 3.20)	.47
Headache	20	1.49 (1.24 to 1.79)	**<.0001**	10	1.29 (0.56 to 2.93)	.55
Hemiparesis	3	2.79 (0.47 to 16.61)	.26	2	—	—
Hemiplegia	4	1.18 (0.24 to 5.87)	.84	4	1.18 (0.24 to 5.87)	.84
Hemorrhage, intracranial	7	0.85 (0.27 to 2.69)	.78	7	0.85 (0.27 to 2.69)	.78
Hypoesthesia	5	1.48 (0.50 to 4.44)	.48	2	—	—
Lethargy	4	0.58 (0.21 to 1.64)	.31	2	—	—
Loss of consciousness	4	0.90 (0.18 to 4.49)	.90	4	0.90 (0.18 to 4.49)	.90
Memory impairment	3	0.89 (0.08 to 10.41)	.92	1	—	—
Nervous system disorder	4	0.72 (0.16 to 3.29)	.67	4	0.72 (0.16 to 3.29)	.67
Neuralgia	6	1.19 (0.32 to 4.35)	.79	4	1.68 (0.34 to 8.34)	.53
Neuropathy, peripheral	10	1.21 (0.66 to 2.22)	.54	2	—	—
Neurotoxicity	3	0.89 (0.19 to 4.17)	.89	NA	—	—
Paresthesia	14	1.20 (0.86 to 1.66)	.28	3	0.24 (0.04 to 1.51)	.13
Peripheral motor neuropathy	8	3.91 (1.14 to 13.42)	**.03**	4	4.27 (0.91 to 20.11)	.07
Polyneuropathy	3	1.65 (0.30 to 9.19)	.57	3	1.24 (0.21 to 7.17)	.81
Presyncope	5	1.80 (0.46 to 7.06)	.40	5	1.50 (0.37 to 6.04)	.56
Sciatica	3	1.33 (0.24 to 7.35)	.74	1	—	—
Seizure	6	1.39 (0.55 to 3.51)	.49	6	1.32 (0.47 to 3.67)	.60
Somnolence	7	1.36 (0.42 to 4.43)	.61	5	1.00 (0.24 to 4.17)	.99
Spinal cord compression	3	1.33 (0.22 to 8.25)	.76	3	1.33 (0.22 to 8.25)	.76
Syncope	12	1.12 (0.49 to 2.56)	.79	11	1.02 (0.43 to 2.42)	.96
Transient ischemic attack	3	1.09 (0.17 to 6.98)	.93	3	1.09 (0.17 to 6.98)	.93
Tremor	4	1.57 (0.38 to 6.53)	.54	1	—	—
**Hematologic adverse events**						
Anemia	20	1.06 (0.70 to 1.62)	.77	13	1.29 (0.69 to 2.42)	.42
Febrile neutropenia	15	0.46 (0.22 to 0.96)	**.04**	15	0.50 (0.25 to 1.02)	.06
Leukopenia	14	0.47 (0.29 to 0.77)	**.002**	5	0.37 (0.10 to 1.35)	.13
Lymphopenia	4	0.69 (0.48 to 0.98)	**.04**	NA	—	—
Neutropenia	17	0.56 (0.31 to 1.01)	**.05**	14	0.73 (0.29 to 1.81)	.49
Pancytopenia	6	0.73 (0.19 to 2.85)	.65	6	0.73 (0.19 to 2.85)	.65
Thrombocytopenia	17	2.07 (1.00 to 4.31)	**.05**	13	1.00 (0.43 to 2.33)	1.00
**Hepatic adverse events**						
Alanine aminotransferase increase	19	2.51 (1.84 to 3.40)	**<.00001**	4	2.01 (0.42 to 9.61)	.38
Aspartate aminotransferase increase	18	2.83 (2.04 to 3.93)	**<.00001**	4	1.94 (0.41 to 9.22)	.41
**Ocular adverse events**						
Blurred vision	7	3.12 (1.02 to 9.55)	**.05**	3	1.03 (0.16 to 6.52)	.98
Cataract	4	3.92 (1.52 to 10.13)	**.005**	1	—	—
Dry eye	9	2.30 (0.86 to 6.10)	.10	NA	—	—
Eye pain	4	9.54 (2.64 to 34.46)	**.0006**	1	—	—
Lacrimation increased	7	1.20 (0.35 to 4.09)	.77	NA	—	—
Photophobia	4	6.84 (2.26 to 20.67)	**.0007**	NA	—	—
**Renal adverse events**						
Acute kidney injury	10	1.38 (0.58 to 3.29)	.46	9	1.49 (0.57 to 3.86)	.42
Dysuria	5	0.60 (0.23 to 1.58)	.30	2	—	—
Hematuria	7	0.63 (0.20 to 1.95)	.42	7	0.63 (0.20 to 1.95)	.42
Kidney failure	7	0.82 (0.29 to 2.34)	.71	7	0.82 (0.29 to 2.34)	.71
Proteinuria	3	0.48 (0.11 to 2.05)	.33	NA	—	—
Urinary retention	6	2.38 (0.66 to 8.63)	.19	4	2.46 (0.49 to 12.23)	.27
Urinary tract obstruction	4	0.53 (0.10 to 2.77)	.45	3	0.26 (0.04 to 1.63)	.15
**Respiratory adverse events**						
Acute respiratory distress syndrome	8	1.28 (0.46 to 3.58)	.64	8	1.28 (0.46 to 3.58)	.64
Acute respiratory failure	4	0.69 (0.14 to 3.27)	.64	4	0.69 (0.14 to 3.27)	.64
Alveolitis, allergic	3	0.53 (0.08 to 3.41)	.51	3	0.53 (0.08 to 3.41)	.51
Asthma	3	0.52 (0.08 to 3.32)	.49	3	0.52 (0.08 to 3.32)	.49
Chronic obstruction pulmonary disease	6	1.78 (0.53 to 5.93)	.35	6	1.78 (0.53 to 5.93)	.35
Cough	19	1.11 (0.95 to 1.29)	.19	5	0.98 (0.24 to 3.93)	.98
Dysphonia	4	0.44 (0.09 to 2.32)	.34	NA	—	—
Dyspnea, exertional	5	1.78 (0.44 to 7.24)	.42	2	—	—
Epistaxis	14	2.45 (1.50 to 4.01)	**.0003**	7	1.61 (0.49 to 5.27)	.43
Hemoptysis	7	1.40 (0.54 to 3.62)	.48	6	0.95 (0.28 to 3.23)	.93
Hypertensive crisis	3	1.68 (0.26 to 10.69)	.58	3	1.68 (0.26 to 10.69)	.58
Hypoxia	11	0.94 (0.41 to 2.13)	.88	9	0.67 (0.25 to 1.80)	.43
Interstitial lung disease	5	1.57 (0.46 to 5.30)	.47	4	1.20 (0.32 to 4.55)	.78
Nasal congestion	5	1.38 (0.37 to 5.19)	.63	NA	—	—
Oropharyngeal pain	14	1.05 (0.87 to 1.27)	.60	NA	—	—
Pleural effusion	15	1.19 (0.50 to 2.82)	.69	14	1.24 (0.57 to 2.68)	.58
Pleurisy	3	1.76 (0.28 to 11.21)	.55	3	1.76 (0.28 to 11.21)	.55
Pleuritic pain	6	0.83 (0.23 to 2.92)	.77	4	0.77 (0.15 to 3.83)	.75
Pneumonia, aspiration	7	1.06 (0.34 to 3.24)	.93	7	1.06 (0.34 to 3.24)	.93
Pneumonitis	15	1.83 (0.79 to 4.22)	.16	15	1.53 (0.71 to 3.27)	.27
Pneumothorax	8	0.82 (0.35 to 1.96)	.66	8	0.88 (0.35 to 2.20)	.78
Productive cough	5	0.99 (0.39 to 2.53)	.98	NA	—	—
Pulmonary embolism	15	0.80 (0.48 to 1.32)	.38	15	0.77 (0.46 to 1.28)	.31
Pulmonary fibrosis	5	1.54 (0.37 to 6.47)	.55	4	1.59 (0.32 to 7.91)	.57
Respiratory failure	12	0.45 (0.17 to 1.16)	.10	12	0.45 (0.17 to 1.16)	.10
Rhinorrhea	6	1.39 (0.85 to 2.26)	.19	NA	—	—

a If the 95% confidence interval does not include “1”, OR > 1 favors the non–antibody-drug conjugate group and OR < 1 favors the antibody-drug conjugate group. The bold values mean statistically significant. NA = not available.

### Hematologic toxicities

Antibody-drug conjugates were associated with a statistically significantly lower risk of all-grade febrile neutropenia (OR = 0.46, 95% CI = 0.22 to 0.96), leukopenia (OR = 0.47, 95% CI = 0.29 to 0.77), lymphopenia (OR = 0.69, 95% CI = 0.48 to 0.98), and neutropenia (OR = 0.56, 95% CI = 0.31 to 1.01) compared with control groups (Figure 2). Antibody-drug conjugates were associated with a higher risk of all-grade thrombocytopenia (OR = 2.07, 95% CI = 1.00 to 4.30) compared with control groups (Supplementary Figure 3, available online). The risk of high-grade hematologic adverse events was not statistically significantly different between these 2 groups (Table 2).

### Hepatic toxicities

Antibody-drug conjugates were associated with a statistically significantly higher risk of all-grade increased alanine aminotransferase (OR = 2.51, 95% CI = 1.84 to 3.40) and increased aspartate aminotransferase (OR = 2.83, 95% CI = 2.04 to 3.93) than antibody-drug conjugate–free therapy (Figure 2;Supplementary Figure 4, available online). Only 4 studies reported incidence of high-grade increased alanine aminotransferase and aspartate aminotransferase, the risk of which was not statistically significantly increased in antibody-drug conjugate groups (Table 2).

### Ocular toxicities

Antibody-drug conjugates were associated with a statistically significantly higher risk of cataract (OR = 3.92, 95% CI = 1.52 to 10.13), eye pain (OR = 9.54, 95% CI = 2.64 to 34.46), photophobia (OR = 6.84, 95% CI = 2.26 to 20.67), and blurred vision (OR = 3.12, 95% CI = 1.02 to 9.55) than seen in control groups (Figure 2;Supplementary Figure 5, available online). The risk of dry eye and increased lacrimation was not statistically significantly different between the 2 groups (Table 2).

### Cardiovascular toxicities

Antibody-drug conjugates were associated with a statistically significantly decreased risk of hot flush (OR = 0.57, 95% CI = 0.37 to 0.88) compared with the control groups (Figure 2). There was no statistically significant difference between antibody-drug conjugate and control groups in the risk of the other 23 all-grade cardiovascular adverse events. The risk of high-grade cardiovascular adverse events was not statistically significantly different between the 2 groups (Table 2).

### Renal toxicities

With regard to adverse events related to the kidney and urinary system, there was no statistically significant difference in the incidence of all-grade and high-grade adverse events between groups with and without antibody-drug conjugates (Table 2).

### Respiratory toxicities

Antibody-drug conjugates were associated with a statistically significantly increased risk of all-grade epistaxis (OR = 2.45, 95% CI = 1.50 to 4.01) (Figure 2). There was no statistically significant difference between antibody-drug conjugate and control groups in the risk of the other 25 all-grade respiratory adverse events. The risk of high-grade respiratory adverse events was not statistically significantly different between the 2 groups (Table 2).

### Gastrointestinal toxicities

Antibody-drug conjugates were associated with a statistically significantly increased risk of all-grade abdominal pain (OR = 1.29, 95% CI = 1.01 to 1.66), upper abdominal pain (OR = 1.53, 95% CI = 1.23 to 1.89), dry mouth (OR = 4.46, 95% CI = 2.56 to 7.78), vomiting (OR = 1.32, 95% CI = 1.01 to 1.74), gingival bleeding (OR = 6.50, 95% CI = 2.80 to 15.12) (Figure 2;Supplementary Figure 6, available online). There was no statistically significant difference between antibody-drug conjugate and control groups in the risk of the other 25 all-grade gastrointestinal adverse events. The risk of high-grade gastrointestinal adverse events was not statistically significantly different between the 2 groups (Table 2).

### Neurologic toxicities

Antibody-drug conjugates were associated with a statistically significantly increased risk of all-grade headache (OR = 1.49, 95% CI = 1.24 to 1.79) and peripheral motor neuropathy (OR = 3.91, 95% CI = 1.14 to 13.42) (Figure 2). There was no statistically significant difference between antibody-drug conjugate and control groups in the risk of the other 31 neurologic adverse events. The risk of high-grade neurologic adverse events was not statistically significantly different between the 2 groups (Table 2).

### Subgroup analyses

For adverse events with larger heterogeneity (*I*^2^ > 50%), we conducted subgroup analyses by type of cancer (solid tumor vs hematologic malignancy) and antibody-drug conjugate regimen (antibody-drug conjugates vs antibody-drug conjugates plus chemotherapy). The risk of all-grade and high-grade febrile neutropenia, all-grade and high-grade neutropenia, and all-grade leukopenia was higher in patients with hematologic malignancies. The risk of all-grade headache was higher in patients with solid tumors, whereas the risk of all-grade peripheral neuropathy was lower in patients with solid tumors. Otherwise, a similar toxicity profile was observed between solid tumor and hematologic malignancy groups (Supplementary Table 1, available online).

Regimens containing antibody-drug conjugates plus chemotherapy were associated with an increased risk of all-grade anemia, febrile neutropenia, leukopenia, and neutropenia as well as high-grade febrile neutropenia and high-grade neutropenia compared with regimens containing antibody-drug conjugates alone. Otherwise, a similar toxicity profile was observed when comparing antibody-drug conjugate with antibody-drug conjugate plus chemotherapy groups (Supplementary Table 2, available online).

## Discussion

In this study, we analyzed the safety of antibody-drug conjugates for cancer treatment using the comprehensive final reports of adverse events occurring in randomized controlled trials. Antibody-drug conjugates were associated with a higher risk of all-grade adverse events, including treatment-related symptoms and toxicities of the gastrointestinal, neurologic, ocular, and hepatic systems. An increased risk of isolated types of adverse events in cardiovascular, hematologic, and respiratory systems was also seen. Conversely, antibody-drug conjugate use was associated with a decreased risk of febrile neutropenia, leukopenia, and neutropenia. The risk of hematologic adverse events, including some high-grade adverse events, was higher in patients with hematologic malignancies and in patients treated with regimens that included antibody-drug conjugates plus chemotherapy. This profile of adverse events in the use of antibody-drug conjugates in patients with malignant neoplasms may provide valuable guidance for patient counseling. A caveat for extrapolating to clinical practice may exist, however, in terms of the heterogeneity of the studies included.

Antibody-drug conjugate regimens were associated with a lower incidence of all-grade febrile neutropenia, leukopenia, lymphopenia, and neutropenia than the non–antibody-drug conjugate regimens; a better hematologic safety profile may be attributable to the tumor-targeted nature of this treatment modality. Neutropenia and febrile neutropenia had been reported as the most common adverse events in previous studies with antibody-drug conjugates using monomethyl auristatin E (MMAE), including pinatuzumab vedotin (36) and polatuzumab vedotin (37). Both trials, however, were single-arm studies and did not compare the incidence of adverse events with conventional anticancer therapy. By comparing with control arms, our result indicates a statistically significantly lower risk of neutropenia and febrile neutropenia in patients treated with antibody-drug conjugates, even in those with an MMAE-conjugated antibody-drug conjugate, such as brentuximab vedotin. A possible explanation for the discrepancy between our results and the previous findings could be the difference in trial design. Alternatively, it has been suggested that in addition to components derived from payload and its metabolite, other components, such as target monoclonal antibodies and linkers may play at least a partial role in antibody-drug conjugate–associated neutropenia and febrile neutropenia. Actually, target monoclonal antibody–mediated neutropenia was reported in a trial with the CD33-specific antibody-drug conjugates gemtuzumab ozogamicin and SGN-33A by directly binding to CD33 on the surface of myeloid progenitor cells (38).

Conversely, there was a statistically significantly higher incidence of thrombocytopenia in patients who received antibody-drug conjugate–based therapy than in those treated with comparator therapy. The higher incidence of epistaxis and gingival bleeding in antibody-drug conjugate–treated patients may be attributable to the thrombocytopenia associated with antibody-drug conjugates. The increased risk of thrombocytopenia was prominent in patients treated with maitansine- and monomethyl auristatin F (MMAF)–conjugated antibody-drug conjugates (39,40). Thrombocytopenia is attributed to impairment of megakaryocyte differentiation (41). Megakaryocytes internalize antibody-drug conjugates in an FcγRII-dependent manner (41) or in macropinocytosis (42), leading to higher exposure to the antibody-drug conjugate in megakaryocytes than in other tissues (43).

Consistent with a previous report showing a clear payload association with ocular toxicities typically induced by antibody-drug conjugates containing DM4 and MMAF4 (44), ocular adverse events observed in included studies, such as cataract, eye pain, photophobia, and blurred vision, are largely associated with DM4-conjugated antibody-drug conjugates (anetumab ravtansine and mirvetuximab soravtansine) and MMAF4-conjugated antibody-drug conjugates (depatuxizumab mafodotin and AGS-16C3F). The pathogenesis of ocular-related adverse events has not been fully elucidated, but the toxicity may be related to the accumulation of the drugs within cells (45,46). Nonetheless, most ocular adverse events were not serious and could be managed with steroid eye drops (47).

In contrast to the ocular adverse events, which were observed only in DM4- or MMAF-conjugated antibody-drug conjugates, hepatic-related adverse events, such as increases in alanine aminotransferase and aspartate aminotransferase levels, were associated with antibody-drug conjugates of different payloads. These results should raise caution among clinicians considering antibody-drug conjugate–based regimens for patients with preexisting liver disease, regardless of malignancy type and antibody-drug conjugate type (48,49).

Several limitations of the present study should be noted. First, the sample sizes of the included studies varied statistically significantly, which may explain the heterogeneity in some results. In addition, small sample size may potentially affect the precision of some estimates. Second, the number of studies was still insufficient to conduct subgroup analysis. For example, only 1 trial evaluated the safety of antibody-drug conjugates such as mirvetuximab soravtansine, and sacituzumab govitecan, making it impossible for subgroup analysis based on each antibody-drug conjugate. In addition, of 20 clinical trials included in our meta-analysis, 16 had an open-label design, which may raise the risk of ascertainment bias. A recent report, however, indicated that there is no evidence of statistically significant bias for patient-reported outcomes based on the absence of blinding in oncology clinical trials (50). Thus, a risk of potential bias resulting from the unblinded design would not affect the between-arm variance in the incidence of the adverse events investigated. Finally, the treatment setting varied in terms of the combination of anticancer drugs and drugs used in the control group. Even though our study showed the overall tendency and risk of adverse events in antibody-drug conjugate–combined regimens, we should keep in mind that heterogeneity exists.

To the best of our knowledge, this is the first study to comprehensively compare the tolerability of antibody-drug conjugate–based regimens with other, standard treatments across multiple malignancies. Our analysis suggests that antibody-drug conjugate–based therapy resulted in a higher incidence of various types of all-grade treatment-related adverse events, including cardiovascular, hepatic, gastrointestinal, neurologic, and ocular toxicity, but a statistically significantly lower risk of all-grade hematologic adverse events, except for thrombocytopenia, which were observed in the antibody-drug conjugate–based groups, compared with the groups not using antibody-drug conjugates. Although our results provide valuable information for clinicians to balance the benefits and risks of treatment options in their decision making, it is crucial to consider the limitations of our study, particularly the heterogeneity of included studies, when applying these findings to clinical practice.

## Supplementary Material

pkad069_Supplementary_Data

## Data Availability

No new data were generated or analyzed in support of this research. The data underlying this study can be shared based on the request to the corresponding author.
