# Supplementary material for: Toxicity profiles of antibody-drug conjugates for anticancer treatment: a systematic review and meta-analysis
Source: JNCI Cancer Spectr. 2023 Sep 26;7(5):pkad069. doi: 10.1093/jncics/pkad069 (PMC10579782; doi:10.1093/jncics/pkad069)

Supplementary Table 1. Summary of subgroup analysis: solid tumor vs hematologic malignancy

	Solid tumor			Hematologic malignancy			Interaction *p*-value
	No. of studies	OR (95% CI)	*p*-value	No. of studies	OR (95% CI)	*p*-value	
**All-grade Treatment-related symptoms**							
Fatigue	14	1.26 (1.04-1.53)	**0.02**	6	1.16 (0.97-1.38)	0.10	0.52
Insomnia	13	1.16 (0.87-1.56)	0.31	5	1.30 (0.93-1.83)	0.12	0.62
Anorexia	13	1.35 (1.00-1.81)	**0.05**	6	1.37 (1.01-1.86□	**0.05**	0.94
Dyspnea	13	1.00 (0.78-1.27)	0.99	6	0.93 (0.53-1.64)	0.81	0.83
Nausea	14	1.54 (1.07-2.21)	**0.02**	6	1.27 (0.81-2.00)	0.30	0.52
Sensory neuropathy	9	1.72 (0.80-3.67)	0.16	4	3.60 (1.46-8.87)	**0.005**	0.22
Constipation	14	1.09 (0.77-1.54)	0.63	6	1.17 (0.83-1.65)	0.36	0.77
Diarrhea	14	0.84 (0.40-1.77)	0.65	6	1.40 (0.74-2.68)	0.30	0.31
**Cardiovascular AEs**							
Hypertension	11	1.24 (0.71-2.16)	0.45	3	1.15 (0.58-2.28)	0.69	0.87
Lymphedema	6	0.55 (0.28-1.11)	0.10	NA	—	—	—
Pericardial effusion	8	1.57 (0.45-5.53)	0.48	1	0.32 (0.01-7.85)	0.48	0.36
**Gastrointestinal AEs**							
Abdominal pain	14	1.22 (0.95-1.57)	0.12	5	1.50 (0.83-2.70)	0.18	0.53
Dry mouth	8	4.46 (2.56-7.78)	**<0.00001**	NA	—	—	—
Stomatitis	13	0.61 (0.32-1.17)	0.14	4	1.06 (0.73-1.53)	0.78	0.15
Vomiting	14	1.33 (0.93-1.92)	0.12	6	1.28 (0.83-2.00)	0.27	0.90
**Neurologic AEs**							
Dysgeusia	11	1.15 (0.63-2.09)	0.65	4	1.01 (0.72-1.42)	0.94	0.72
Headache	14	1.77 (1.49-2.11)	**<0.00001**	6	1.05 (0.85-1.30)	0.66	**0.0002**
Neuropathy peripheral	8	1.05 (0.55-2.02)	0.88	2	2.41 (1.81-3.21)	**<0.00001**	**0.02**
Paresthesia	10	1.12 (0.77-1.64)	0.55	4	1.72 (0.70-4.27)	0.24	0.39
Peripheral motor neuropathy	4	4.10 (0.91-18.4)	0.07	4	3.97 (0.68-23.03)	0.12	0.98
**Hematologic AEs**							
Anemia	14	1.09 (0.64-1.86)	0.76	6	1.02 (0.48-2.16)	0.96	0.89
Febrile neutropenia	11	0.27 (0.10-0.73)	**0.01**	4	1.03 (0.33-3.15)	0.97	**0.08**
Leukopenia	9	0.23 (0.13-0.41)	**<0.00001**	5	0.93 (0.63-1.38)	0.73	**<0.0001**
Neutropenia	11	0.32 (0.12-0.82)	**0.02**	6	1.37 (0.84-2.23)	0.21	**0.007**
Thrombocytopenia	12	2.59 (0.88-7.62)	0.08	5	1.47 (0.71-3.07)	0.30	0.40
**Hepatic AEs**							
ALT increased	14	2.80 (1.95-4.02)	**<0.00001**	5	1.86 (1.02-3.37)	**0.04**	0.25
AST increased	14	3.06 (2.04-4.58)	**<0.00001**	4	2.40 (1.48-3.90)	**0.0004**	0.46
**Ocular AEs**							
Vision blurred	7	3.12 (1.02-9.55)	**0.05**	NA	—	—	—
**Respiratory AEs**							
Epistaxis	13	2.54 (1.50-4.31)	**0.0005**	1	1.71 (0.84-3.51)	0.14	0.38
Pleural effusion	13	1.26 (0.50-3.16)	0.63	2	0.64 (0.13-3.11)	0.58	0.47
**High-grade Hematologic AEs**							
Febrile neutropenia	11	0.27 (0.10-0.74)	**0.01**	4	1.18 (0.51-2.75)	0.70	**0.03**
Neutropenia	9	0.37 (0.11-1.28)	0.12	5	2.17 (0.96-4.90)	0.06	**0.02**

Abbreviations: AE, adverse event; OR, odds ratio; CI, confidence interval.

Supplementary Table 2. Summary of subgroup analysis: ADC vs ADC + chemo

	ADC			ADC + chemo			Interaction *p*-value
	No. of studies	OR (95% CI)	*p*-value	No. of studies	OR (95% CI)	*p*-value	
**All-grade Treatment-related symptoms**							
Fatigue	15	1.21 (1.01-1.45)	**0.04**	7	1.28 (1.00-1.62)	**0.05**	0.74
Insomnia	14	1.12 (0.85-1.46)	0.42	6	1.33 (0.95-1.85)	0.09	0.42
Anorexia	14	1.40 (1.05-1.87)	**0.02**	6	1.24 (0.98-1.57□	0.07	0.53
Dyspnea	14	0.91 (0.71-1.18)	0.48	7	1.03 (0.66-1.61)	0.90	0.64
Nausea	15	1.51 (1.02-2.26)	**0.04**	7	1.27 (0.93-1.72)	0.13	0.49
Sensory neuropathy	10	2.05 (0.97-4.33)	0.06	4	1.87 (0.81-4.33)	0.14	0.87
Constipation	15	1.02 (0.69-1.49)	0.94	7	1.20 (1.04-1.40)	**0.01**	0.41
Diarrhea	15	0.87 (0.40-1.88)	0.72	7	1.26 (0.86-1.84)	0.23	0.39
**Cardiovascular AEs**							
Hypertension	12	1.19 (0.71-1.98)	0.52	4	1.68 (0.90-3.13)	0.10	0.40
Lymphedema	6	0.57 (0.29-1.12)	0.10	1	16.8 (0.96-291.5)	**0.05**	**0.02**
Pericardial effusion	8	0.91 (0.23-3.68)	0.90	2	2.51 (0.10-62.26)	0.58	0.57
**Gastrointestinal AEs**							
Abdominal pain	14	1.22 (0.92-1.61)	0.17	7	1.48 (1.03-2.11)	**0.03**	0.40
Dry mouth	8	4.53 (2.59-7.94)	**<0.00001**	1	3.67 (1.94-6.92)	**<0.0001**	0.62
Stomatitis	12	0.57 (0.29-1.12)	0.11	6	0.97 (0.68-1.39)	0.87	0.18
Vomiting	15	1.37 (0.92-2.03)	0.12	7	1.21 (0.92-1.60)	0.17	0.62
**Neurologic AEs**							
Dysgeusia	12	1.23 (0.65-2.30)	0.52	4	0.92 (0.70-1.20)	0.53	0.41
Headache	15	1.64 (1.36-1.99)	**<0.00001**	7	1.31 (1.00-1.72)	**0.05**	0.18
Neuropathy peripheral	9	1.06 (0.54-2.08)	0.87	2	1.21 (0.31-4.68)	0.78	0.86
Paraesthesia	11	1.16 (0.76-1.79)	0.49	4	1.11 (0.70-1.78)	0.65	0.89
Peripheral motor neuropathy	5	9.31 (3.64-23.82)	**<0.00001**	3	2.38 (0.31-18.11)	0.40	0.23
**Hematologic AEs**							
Anemia	15	0.90 (0.54-1.50)	0.68	7	1.67 (1.02-2.75)	**0.04**	**0.09**
Febrile neutropenia	10	0.26 (0.11-0.64)	**0.003**	6	0.99 (0.46-2.15)	0.99	**0.03**
Leukopenia	9	0.31 (0.15-0.65)	**0.002**	7	0.88 (0.66-1.17)	0.37	**0.01**
Neutropenia	12	0.33 (0.13-0.85)	**0.02**	6	1.27 (0.78-2.09)	0.34	**0.01**
Thrombocytopenia	12	2.21 (0.77-6.36)	0.14	7	2.03 (0.83-4.95)	0.12	0.91
**Hepatic AEs**							
ALT increased	14	2.26 (1.54-3.30)	**<0.0001**	7	3.29 (2.26-4.79)	**<0.00001**	0.17
AST increased	14	2.74 (1.77-4.24)	**<0.00001**	6	3.25 (2.40-4.41)	**<0.00001**	0.53
**Ocular AEs**							
Vision blurred	6	3.48 (1.05-11.55)	**0.04**	3	2.29 (0.34-15.68)	0.40	0.72
**Respiratory AEs**							
Epistaxis	11	2.58 (1.51-4.42)	**0.0005**	4	2.96 (2.09-4.20)	**<0.00001**	0.68
Pleural effusion	12	0.90 (0.39-2.07)	0.81	4	2.42 (0.44-13.15)	0.31	0.31
**High-grade Hematologic AEs**							
Febrile neutropenia	10	0.27 (0.10-0.72)	**0.009**	6	0.92 (0.41-2.11)	0.85	**0.06**
Neutropenia	9	0.37 (0.12-1.17)	0.09	6	1.51 (0.52-4.38)	0.45	**0.08**

Abbreviations: ADC, antibody-drug conjugate; AE, adverse event; OR, odds ratio; CI, confidence interval.

Supplementary Figure 1. Assessment of risk bias

A Traffic light plot

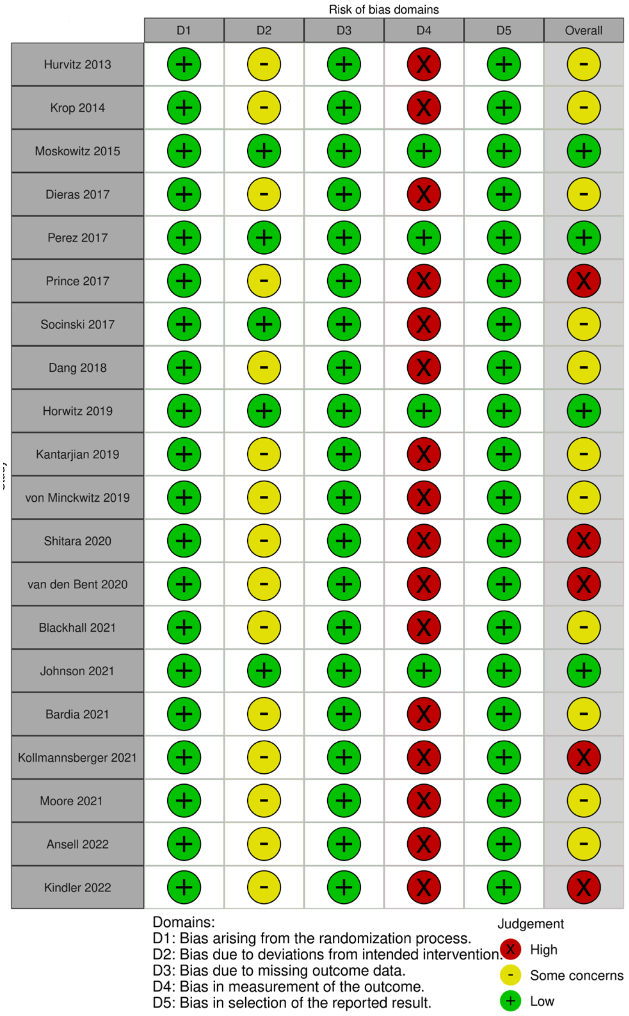

B Summary plot

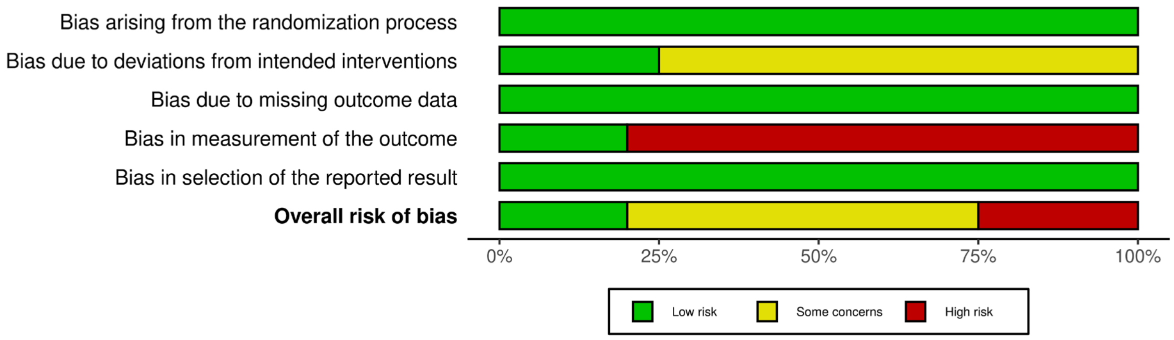

Supplementary Figure 2. Forest plot of incidence of treatment -related symptoms

A Fatigue

	ADC		Control			Odds Ratio
Study or Subgroup	Events	Total	Events	Total	Weight	M-H, Random, 95% CI
Ansell,[34] 2022	211	662	211	659	8.1%	0.99 [0.79, 1.25]
Bardia,[28] 2021	133	258	89	224	6.3%	1.61 [1.12, 2.32]
Blackhall,[30] 2021	71	287	35	129	5.0%	0.88 [0.55, 1.41]
Dang,[23] 2018	56	164	43	167	4.9%	1.50 [0.93, 2.40]
Dieras,[18] 2017	181	490	145	488	7.6%	1.39 [1.06, 1.81]
Horwitz,[24] 2019	83	223	80	226	6.0%	1.08 [0.74, 1.59]
Hurvitz,[16] 2013	34	69	30	66	3.2%	1.17 [0.59, 2.29]
Johnson,[31] 2021	93	368	60	373	6.3%	1.76 [1.23, 2.54]
Kantarjian,[25] 2019	42	164	24	143	4.1%	1.71 [0.97, 2.99]
Kindler,[35] 2022	61	163	23	72	3.9%	1.27 [0.71, 2.29]
Kollmannsberger,[32] 2021	36	66	38	65	3.1%	0.85 [0.43, 1.70]
Krop,[18] 2014	133	497	48	184	6.0%	1.04 [0.70, 1.52]
Moore,[33] 2021	83	243	39	109	4.9%	0.93 [0.58, 1.49]
Moskowitz,[17] 2015	40	167	29	160	4.3%	1.42 [0.83, 2.43]
Perez,[20] 2017	252	727	131	353	7.7%	0.90 [0.69, 1.17]
Prince,[21] 2017	19	66	18	62	2.7%	0.99 [0.46, 2.12]
Shitara,[27] 2020	28	125	16	62	3.0%	0.83 [0.41, 1.68]
Socinsk,[22] 2017	47	94	14	47	2.8%	2.36 [1.12, 4.96]
van den Bent,[28] 2020	60	172	5	21	1.6%	1.71 [0.60, 4.91]
von Minckwitz,[26] 2019	366	740	243	720	8.4%	1.92 [1.56, 2.37]
**Total (95% CI)**		**5745**		**4330**	**100.0%**	**1.25 [1.08, 1.45]**
Total events	2029		1321			

Heterogeneity: $Tau^2 = 0.06$; $Chi^2 = 44.33$, df = 19 (P = 0.0008); $I^2 = 57\%$
Test for overall effect: Z = 3.03 (P = 0.002)

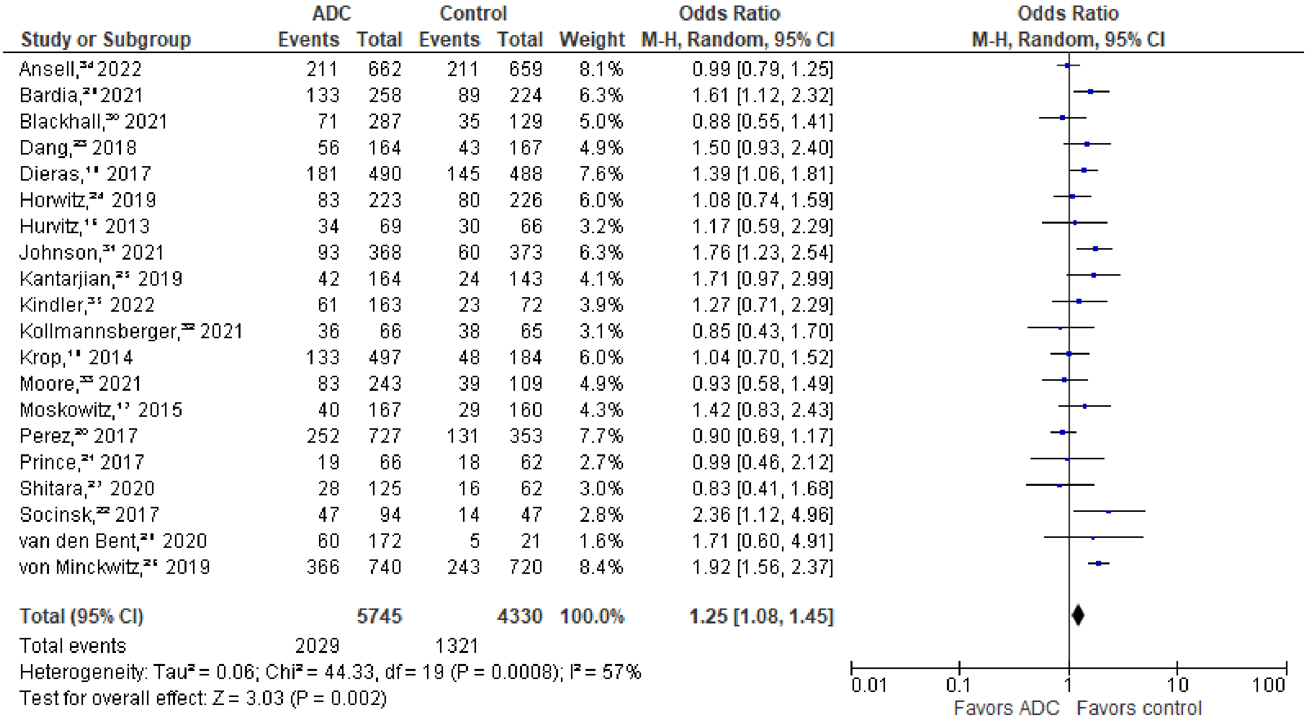

B Anorexia

	ADC		Control			Odds Ratio
Study or Subgroup	Events	Total	Events	Total	Weight	M-H, Random, 95% CI
Ansell,[34] 2022	118	662	76	659	7.8%	1.66 [1.22, 2.27]
Bardia,[28] 2021	71	258	46	224	6.9%	1.47 [0.96, 2.24]
Blackhall,[30] 2021	73	287	36	129	6.5%	0.88 [0.55, 1.41]
Dang,[23] 2018	30	164	33	167	5.9%	0.91 [0.52, 1.57]
Dieras,[18] 2017	105	490	117	488	7.9%	0.86 [0.64, 1.17]
Horwitz,[24] 2019	54	223	47	226	6.7%	1.22 [0.78, 1.90]
Hurvitz,[16] 2013	13	69	11	66	3.7%	1.16 [0.48, 2.81]
Johnson,[31] 2021	6	368	2	373	1.5%	3.07 [0.62, 15.33]
Kantarjian,[25] 2019	19	164	18	143	4.8%	0.91 [0.46, 1.81]
Kindler,[35] 2022	59	163	18	72	5.3%	1.70 [0.91, 3.17]
Kollmannsberger,[32] 2021	14	66	24	65	4.3%	0.46 [0.21, 1.00]
Krop,[18] 2014	83	497	25	184	6.4%	1.28 [0.79, 2.07]
Moore,[33] 2021	60	243	17	109	5.5%	1.77 [0.98, 3.21]
Moskowitz,[17] 2015	20	167	9	160	4.0%	2.28 [1.01, 5.18]
Perez,[20] 2017	168	727	77	353	7.9%	1.08 [0.79, 1.46]
Prince,[21] 2017	10	66	3	62	2.1%	3.51 [0.92, 13.43]
Shitara,[27] 2020	26	125	2	62	1.8%	7.88 [1.81, 34.39]
Socinsk,[22] 2017	32	94	16	47	4.5%	1.00 [0.48, 2.09]
van den Bent,[28] 2020	15	172	0	21	0.6%	4.23 [0.24, 73.30]
von Minckwitz,[26] 2019	62	740	16	720	5.8%	4.02 [2.30, 7.04]
**Total (95% CI)**		**5745**		**4330**	**100.0%**	**1.36 [1.09, 1.69]**
Total events	1038		593			

Heterogeneity: $Tau^2 = 0.13$; $Chi^2 = 53.05$, df = 19 (P < 0.0001); $I^2 = 64\%$
Test for overall effect: Z = 2.74 (P = 0.006)

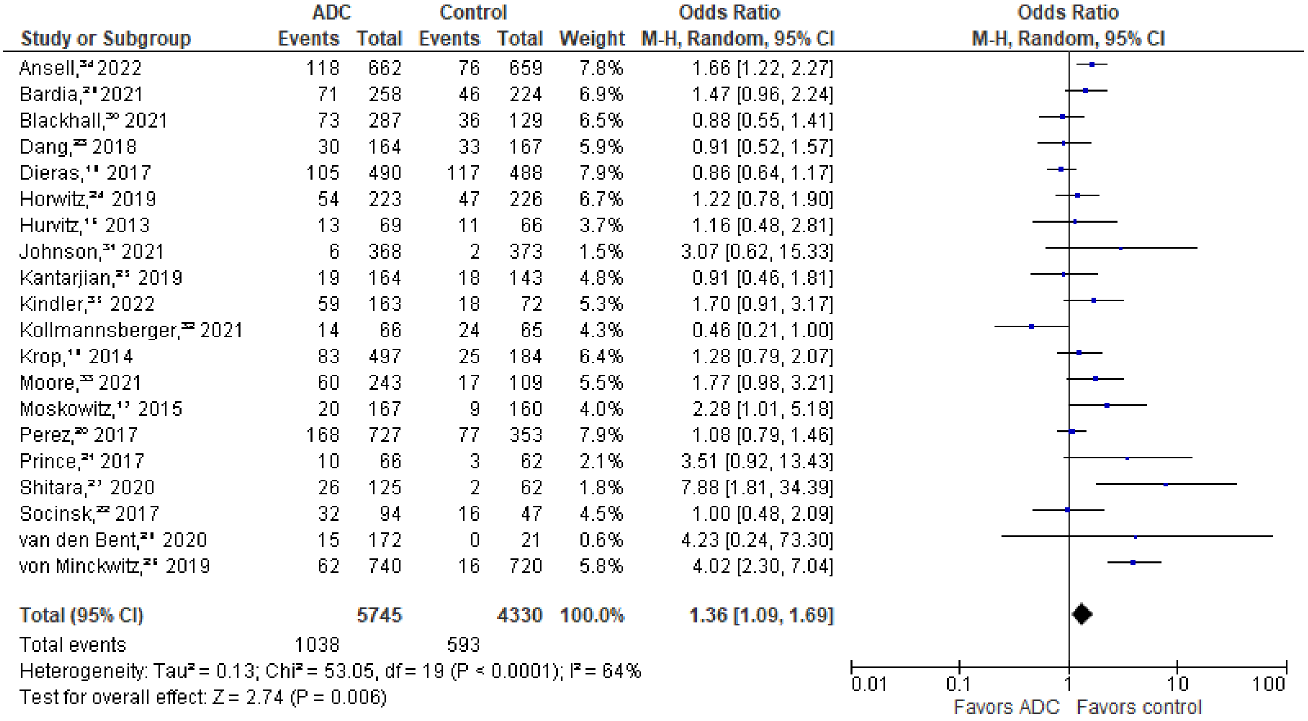

C Nausea

Study or Subgroup	ADC Events	ADC Total	Control Events	Control Total	Weight	Odds Ratio M-H, Random, 95% CI
Ansell,[34] 2022	353	662	372	659	5.7%	0.88 [0.71, 1.09]
Bardia,[28] 2021	162	258	68	224	5.4%	3.87 [2.65, 5.66]
Blackhall,[30] 2021	67	287	40	129	5.2%	0.68 [0.43, 1.08]
Dang,[23] 2018	50	164	55	167	5.2%	0.89 [0.56, 1.42]
Dieras,[18] 2017	204	490	227	488	5.7%	0.82 [0.64, 1.06]
Horwitz,[24] 2019	115	223	95	226	5.4%	1.47 [1.01, 2.13]
Hurvitz,[15] 2013	34	69	29	66	4.5%	1.24 [0.63, 2.44]
Johnson,[31] 2021	80	368	51	373	5.4%	1.75 [1.19, 2.58]
Kantarjian,[25] 2019	54	164	68	143	5.2%	0.54 [0.34, 0.86]
Kindler,[35] 2022	68	163	24	72	4.8%	1.43 [0.80, 2.56]
Kollmannsberger,[32] 2021	31	66	26	65	4.5%	1.33 [0.66, 2.66]
Krop,[18] 2014	160	497	42	184	5.4%	1.61 [1.08, 2.38]
Moore,[33] 2021	133	243	47	109	5.2%	1.59 [1.01, 2.52]
Moskowitz,[17] 2015	38	167	13	160	4.5%	3.33 [1.70, 6.53]
Perez,[20] 2017	367	727	131	353	5.7%	1.73 [1.33, 2.24]
Prince,[21] 2017	24	66	9	62	3.9%	3.37 [1.41, 8.00]
Shitara,[27] 2020	80	125	29	62	4.7%	2.02 [1.09, 3.75]
Socinsk,[22] 2017	40	94	17	47	4.4%	1.31 [0.64, 2.69]
van den Bent,[28] 2020	29	172	6	21	3.5%	0.51 [0.18, 1.42]
von Minckwitz,[26] 2019	309	740	94	720	5.7%	4.77 [3.68, 6.20]
**Total (95% CI)**		**5745**		**4330**	**100.0%**	**1.46 [1.09, 1.97]**
Total events	2398		1443			

Heterogeneity: $Tau^2 = 0.39$; $Chi^2 = 195.43$, df = 19 ($P < 0.00001$); $I^2 = 90\%$

Test for overall effect: Z = 2.50 (P = 0.01)

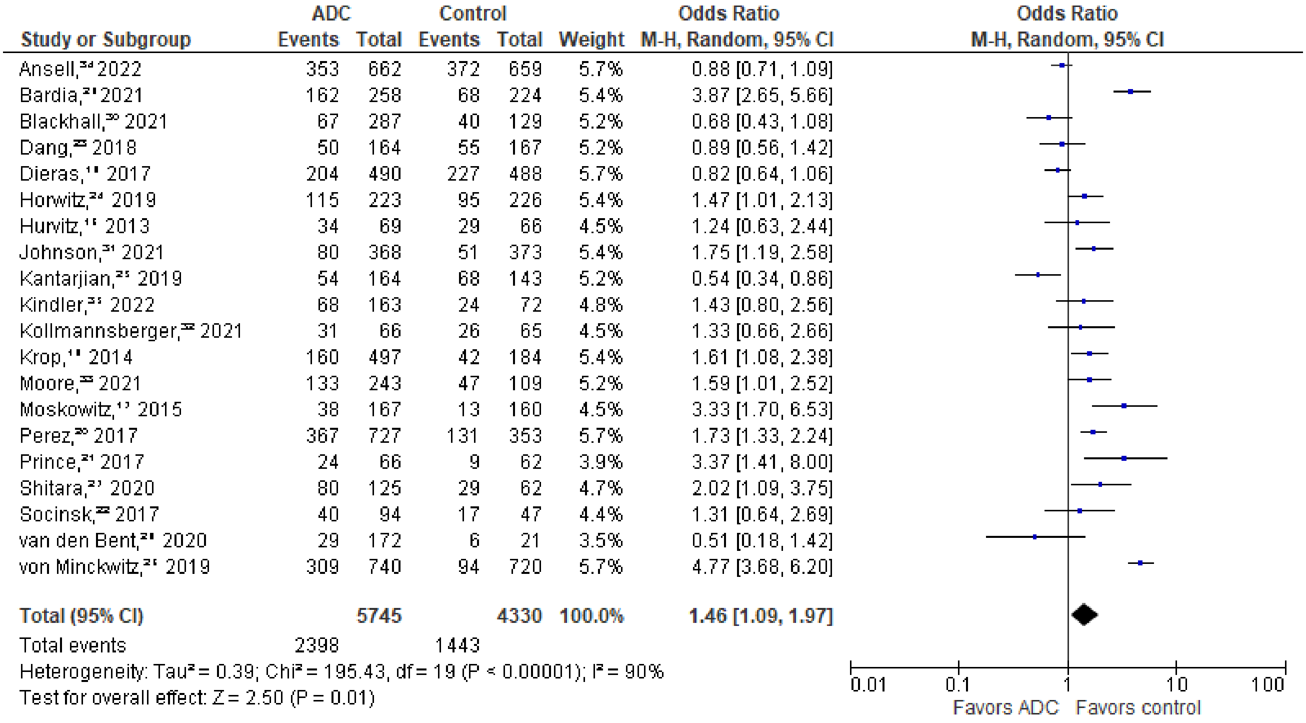

D Sensory neuropathy

Study or Subgroup	ADC Events	ADC Total	Control Events	Control Total	Weight	Odds Ratio M-H, Random, 95% CI
Ansell,[34] 2022	191	662	111	659	10.2%	2.00 [1.54, 2.61]
Dieras,[18] 2017	36	490	27	488	9.6%	1.35 [0.81, 2.27]
Horwitz,[24] 2019	114	223	108	226	10.0%	1.14 [0.79, 1.65]
Hurvitz,[15] 2013	5	69	14	66	7.4%	0.29 [0.10, 0.86]
Kindler,[35] 2022	20	163	0	72	2.7%	20.71 [1.24, 347.38]
Kollmannsberger,[32] 2021	5	66	2	65	5.2%	2.58 [0.48, 13.81]
Moore,[33] 2021	25	243	11	109	8.7%	1.02 [0.48, 2.16]
Moskowitz,[17] 2015	95	167	25	160	9.5%	7.13 [4.21, 12.05]
Perez,[20] 2017	93	727	71	353	10.0%	0.58 [0.42, 0.82]
Prince,[21] 2017	31	66	1	62	4.2%	54.03 [7.07, 413.12]
Shitara,[27] 2020	4	125	2	62	5.1%	0.99 [0.18, 5.57]
Socinsk,[22] 2017	59	94	4	47	7.3%	18.12 [5.99, 54.80]
von Minckwitz,[26] 2019	138	740	50	720	10.0%	3.07 [2.18, 4.32]
**Total (95% CI)**		**3835**		**3089**	**100.0%**	**2.18 [1.27, 3.76]**
Total events	816		426			

Heterogeneity: $Tau^2 = 0.73$; $Chi^2 = 130.85$, df = 12 ($P < 0.00001$); $I^2 = 91\%$

Test for overall effect: Z = 2.82 (P = 0.005)

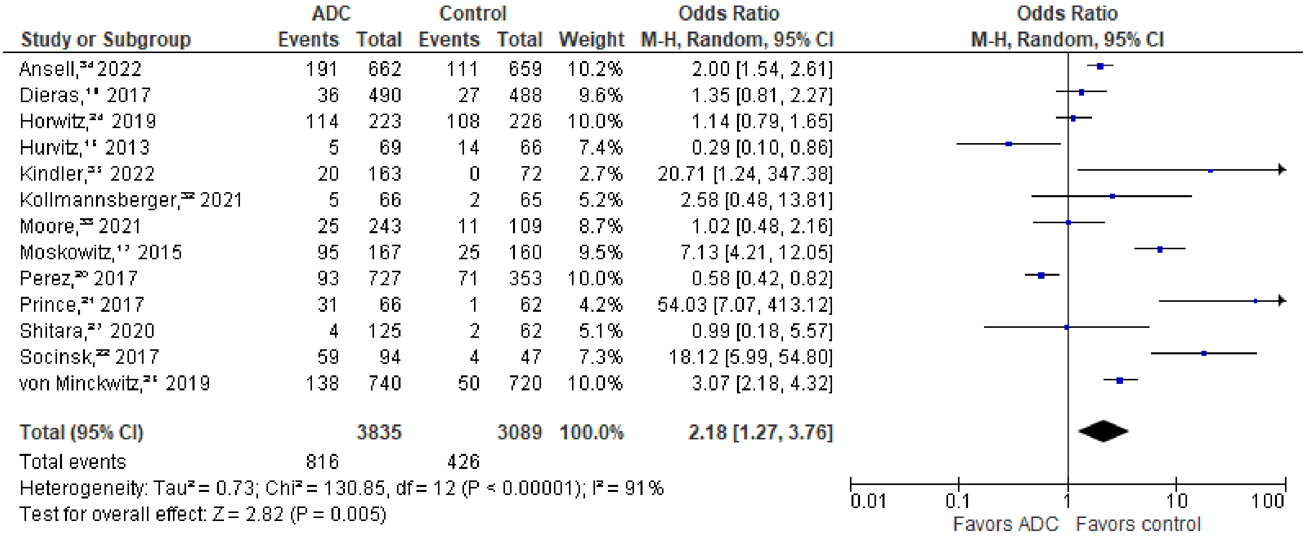

Supplementary Figure 3. Forest plot of incidence of hematologic toxicities

A Febrile neutropenia

Study or Subgroup	ADC Events	ADC Total	Control Events	Control Total	Weight	Odds Ratio M-H, Random, 95% CI
Ansell,[34] 2022	114	662	43	659	9.9%	2.98 [2.06, 4.31]
Bardia,[28] 2021	13	258	4	224	8.2%	2.92 [0.94, 9.08]
Blackhall,[30] 2021	4	287	11	129	8.1%	0.15 [0.05, 0.49]
Dang,[23] 2018	5	164	7	167	8.1%	0.72 [0.22, 2.31]
Dieras,[18] 2017	0	490	2	488	3.7%	0.20 [0.01, 4.14]
Horwitz,[24] 2019	48	223	35	226	9.8%	1.50 [0.92, 2.42]
Hurvitz,[15] 2013	0	69	6	66	3.9%	0.07 [0.00, 1.21]
Johnson,[31] 2021	2	368	3	373	6.3%	0.67 [0.11, 4.06]
Kantarjian,[25] 2019	46	164	79	143	9.8%	0.32 [0.20, 0.51]
Kindler,[35] 2022	1	163	4	72	5.3%	0.10 [0.01, 0.96]
Krop,[18] 2014	0	497	7	184	4.0%	0.02 [0.00, 0.42]
Moore,[33] 2021	0	243	1	109	3.5%	0.15 [0.01, 3.68]
Perez,[20] 2017	0	727	13	353	4.1%	0.02 [0.00, 0.29]
Shitara,[27] 2020	7	125	3	62	7.5%	1.17 [0.29, 4.68]
Socinsk,[22] 2017	6	94	5	47	7.9%	0.57 [0.17, 1.98]
**Total (95% CI)**		**4534**		**3302**	**100.0%**	**0.46 [0.22, 0.96]**
Total events	246		223			

Heterogeneity: $Tau^2 = 1.38$; $Chi^2 = 98.80$, df = 14 ($P < 0.00001$); $I^2 = 86\%$

Test for overall effect: Z = 2.08 (P = 0.04)

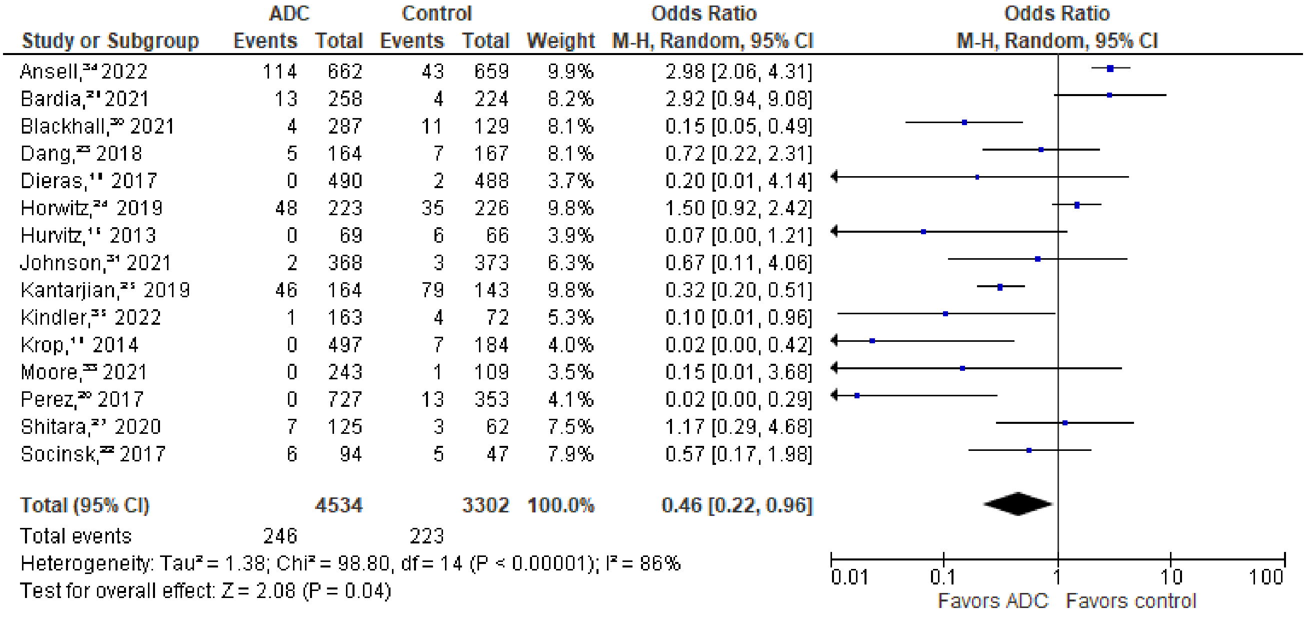

B Leukopenia

Study or Subgroup	ADC Events	ADC Total	Control Events	Control Total	Weight	Odds Ratio M-H, Random, 95% CI
Ansell,[34] 2022	42	662	37	659	10.9%	1.14 [0.72, 1.80]
Blackhall,[30] 2021	4	287	26	129	7.5%	0.06 [0.02, 0.16]
Dang,[23] 2018	37	164	54	167	10.7%	0.61 [0.37, 0.99]
Horwitz,[24] 2019	19	223	16	226	9.6%	1.22 [0.61, 2.44]
Hurvitz,[15] 2013	7	69	17	66	8.2%	0.33 [0.13, 0.85]
Johnson,[31] 2021	0	368	1	373	1.9%	0.34 [0.01, 8.30]
Kantarjian,[25] 2019	48	164	54	143	10.8%	0.68 [0.42, 1.10]
Kindler,[35] 2022	2	163	5	72	5.0%	0.17 [0.03, 0.88]
Krop,[18] 2014	9	497	11	184	8.5%	0.29 [0.12, 0.71]
Moore,[33] 2021	9	243	16	109	8.8%	0.22 [0.10, 0.52]
Moskowitz,[17] 2015	9	167	3	160	6.3%	2.98 [0.79, 11.22]
Perez,[20] 2017	0	727	1	353	1.9%	0.16 [0.01, 3.97]
Socinsk,[22] 2017	10	94	6	47	7.5%	0.81 [0.28, 2.39]
van den Bent,[28] 2020	1	172	1	21	2.4%	0.12 [0.01, 1.94]
**Total (95% CI)**		**4000**		**2709**	**100.0%**	**0.47 [0.29, 0.77]**
Total events	197		248			

Heterogeneity: $Tau^2 = 0.51$; $Chi^2 = 50.17$, df = 13 ($P < 0.00001$); $I^2 = 74\%$

Test for overall effect: Z = 3.03 (P = 0.002)

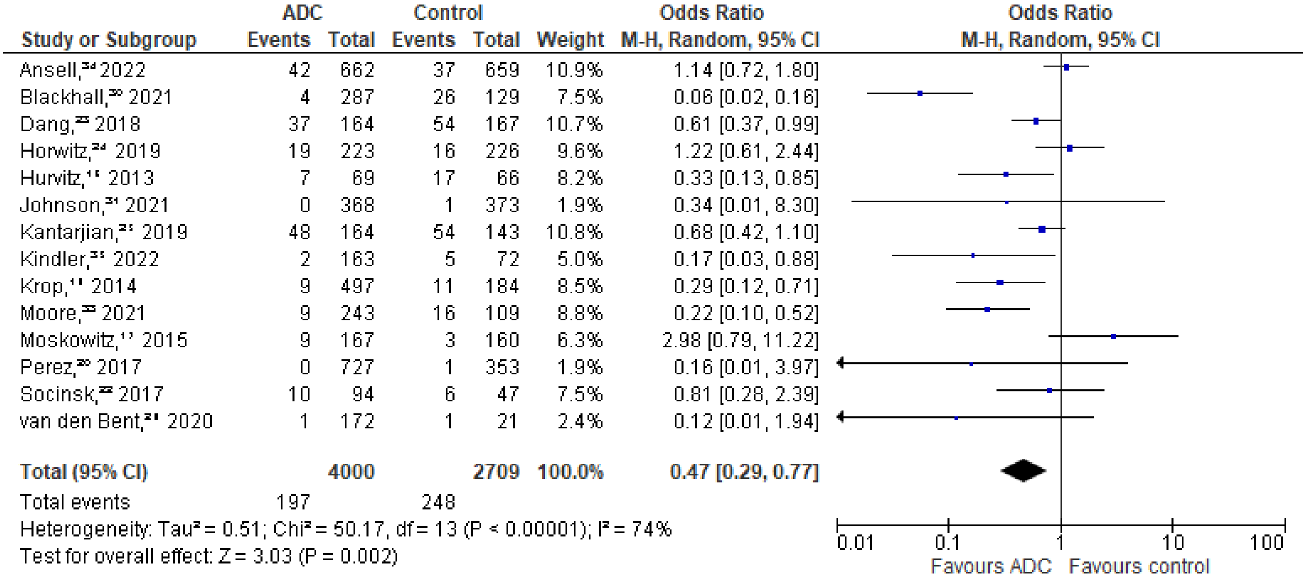

C Lymphopenia

Study or Subgroup	ADC Events	ADC Total	Control Events	Control Total	Weight	Odds Ratio M-H, Random, 95% CI
Dang,[23] 2018	28	164	39	167	42.3%	0.68 [0.39, 1.16]
Kantarjian,[25] 2019	31	164	36	143	42.1%	0.69 [0.40, 1.19]
Moore,[33] 2021	8	243	3	109	6.9%	1.20 [0.31, 4.62]
van den Bent,[28] 2020	17	172	4	21	8.7%	0.47 [0.14, 1.55]
**Total (95% CI)**		**743**		**440**	**100.0%**	**0.69 [0.48, 0.98]**
Total events	84		82			

Heterogeneity: $Tau^2 = 0.00$; $Chi^2 = 1.07$, df = 3 (P = 0.78); $I^2 = 0\%$

Test for overall effect: Z = 2.08 (P = 0.04)

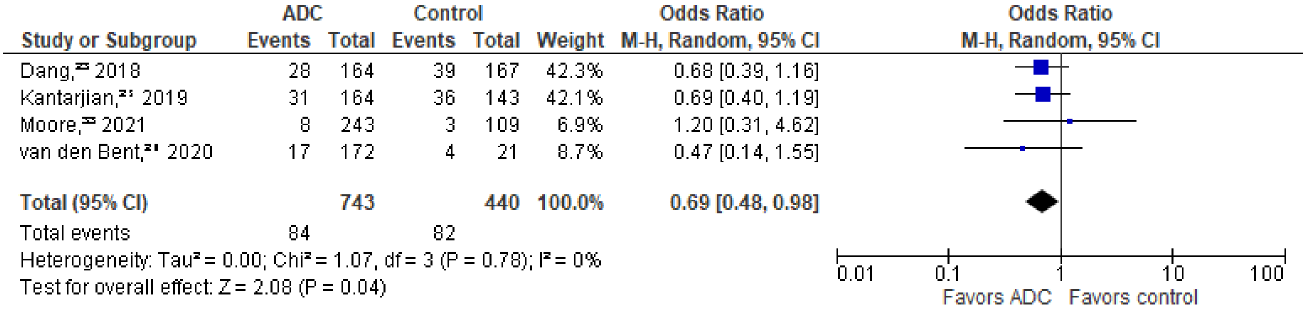

D Neutropenia

Study or Subgroup	ADC Events	ADC Total	Control Events	Control Total	Weight	Odds Ratio M-H, Random, 95% CI
Ansell,[34] 2022	393	662	295	659	6.7%	1.80 [1.45, 2.24]
Bardia,[28] 2021	113	258	57	224	6.6%	2.28 [1.55, 3.37]
Blackhall,[30] 2021	14	287	56	129	6.3%	0.07 [0.04, 0.13]
Dang,[23] 2018	57	164	82	167	6.5%	0.55 [0.35, 0.86]
Dieras,[18] 2017	37	490	44	488	6.5%	0.82 [0.52, 1.30]
Horwitz,[24] 2019	95	223	87	226	6.6%	1.19 [0.81, 1.73]
Hurvitz,[16] 2013	11	69	43	66	6.0%	0.10 [0.04, 0.23]
Johnson,[31] 2021	33	368	10	373	6.1%	3.58 [1.74, 7.37]
Kantarjian,[25] 2019	81	164	66	143	6.5%	1.14 [0.73, 1.78]
Kindler,[35] 2022	4	163	38	72	5.5%	0.02 [0.01, 0.07]
Krop,[18] 2014	36	497	41	184	6.5%	0.27 [0.17, 0.44]
Moore,[33] 2021	16	243	43	109	6.3%	0.11 [0.06, 0.20]
Moskowitz,[17] 2015	59	167	19	160	6.4%	4.05 [2.28, 7.20]
Perez,[20] 2017	0	727	5	353	2.5%	0.04 [0.00, 0.79]
Prince,[21] 2017	5	66	4	62	4.9%	1.19 [0.30, 4.64]
Shitara,[27] 2020	3	125	2	62	4.1%	0.74 [0.12, 4.53]
Socinsk,[22] 2017	56	94	25	47	6.2%	1.30 [0.64, 2.63]
**Total (95% CI)**		**4767**		**3524**	**100.0%**	**0.56 [0.31, 1.01]**
Total events	1013		917			

Heterogeneity: $Tau^2 = 1.31$; $Chi^2 = 314.45$, df = 16 ($P < 0.00001$); $I^2 = 95\%$
Test for overall effect: Z = 1.93 (P = 0.05)

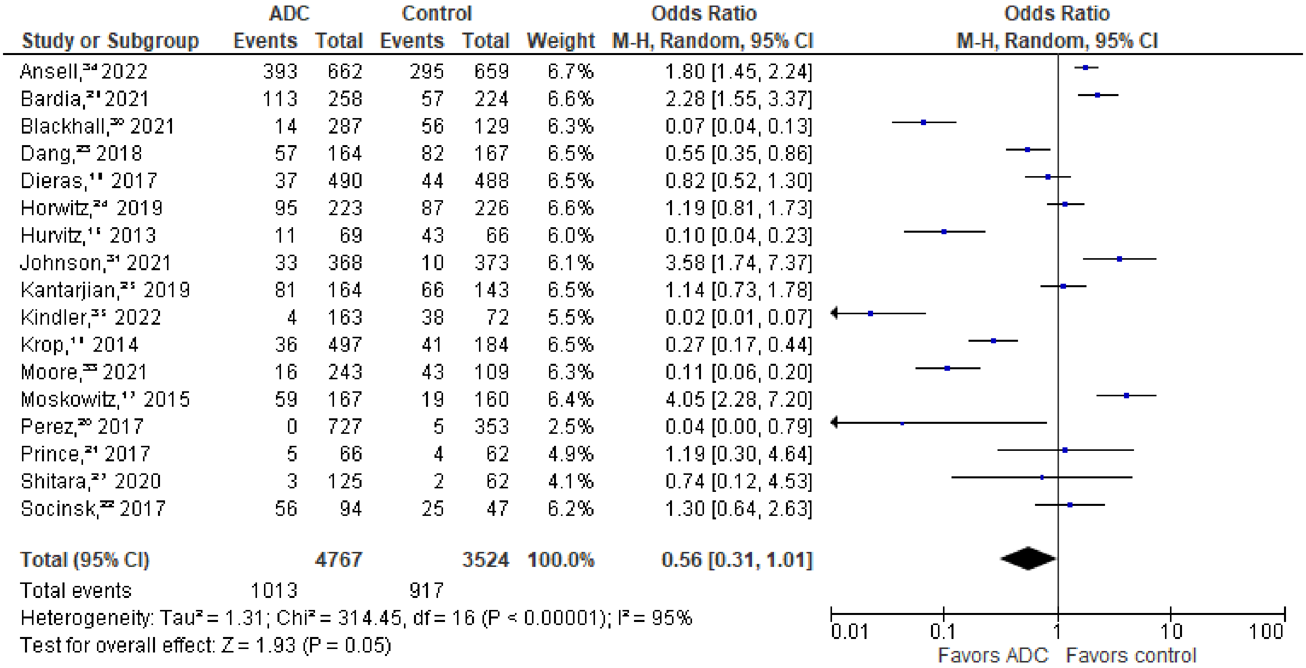

E Thrombocytopenia

Study or Subgroup	ADC Events	ADC Total	Control Events	Control Total	Weight	Odds Ratio M-H, Random, 95% CI
Ansell,[34] 2022	1	662	1	659	3.5%	1.00 [0.06, 15.95]
Bardia,[28] 2021	10	258	14	224	6.5%	0.60 [0.26, 1.39]
Blackhall,[30] 2021	44	287	55	129	6.9%	0.24 [0.15, 0.39]
Dang,[23] 2018	103	164	66	167	6.9%	2.58 [1.66, 4.03]
Dieras,[18] 2017	154	490	14	488	6.8%	15.52 [8.82, 27.29]
Horwitz,[24] 2019	22	223	15	226	6.7%	1.54 [0.78, 3.05]
Hurvitz,[16] 2013	19	69	4	66	6.1%	5.89 [1.88, 18.43]
Johnson,[31] 2021	69	368	14	373	6.8%	5.92 [3.27, 10.73]
Kantarjian,[25] 2019	81	164	87	143	6.9%	0.63 [0.40, 0.99]
Kindler,[35] 2022	8	163	0	72	3.4%	7.93 [0.45, 139.20]
Kollmannsberger,[32] 2021	7	66	0	65	3.4%	16.51 [0.92, 295.38]
Krop,[18] 2014	99	497	7	184	6.6%	6.29 [2.86, 13.81]
Moore,[33] 2021	27	243	17	109	6.7%	0.68 [0.35, 1.30]
Moskowitz,[17] 2015	12	167	5	160	6.2%	2.40 [0.83, 6.97]
Perez,[20] 2017	117	727	0	353	3.5%	136.07 [8.44, 2195.09]
Socinsk,[22] 2017	33	94	21	47	6.7%	0.67 [0.33, 1.37]
van den Bent,[28] 2020	26	172	8	21	6.3%	0.29 [0.11, 0.77]
**Total (95% CI)**		**4814**		**3486**	**100.0%**	**2.07 [1.00, 4.31]**
Total events	832		328			

Heterogeneity: $Tau^2 = 1.95$; $Chi^2 = 239.46$, df = 16 ($P < 0.00001$); $I^2 = 93\%$
Test for overall effect: Z = 1.95 (P = 0.05)

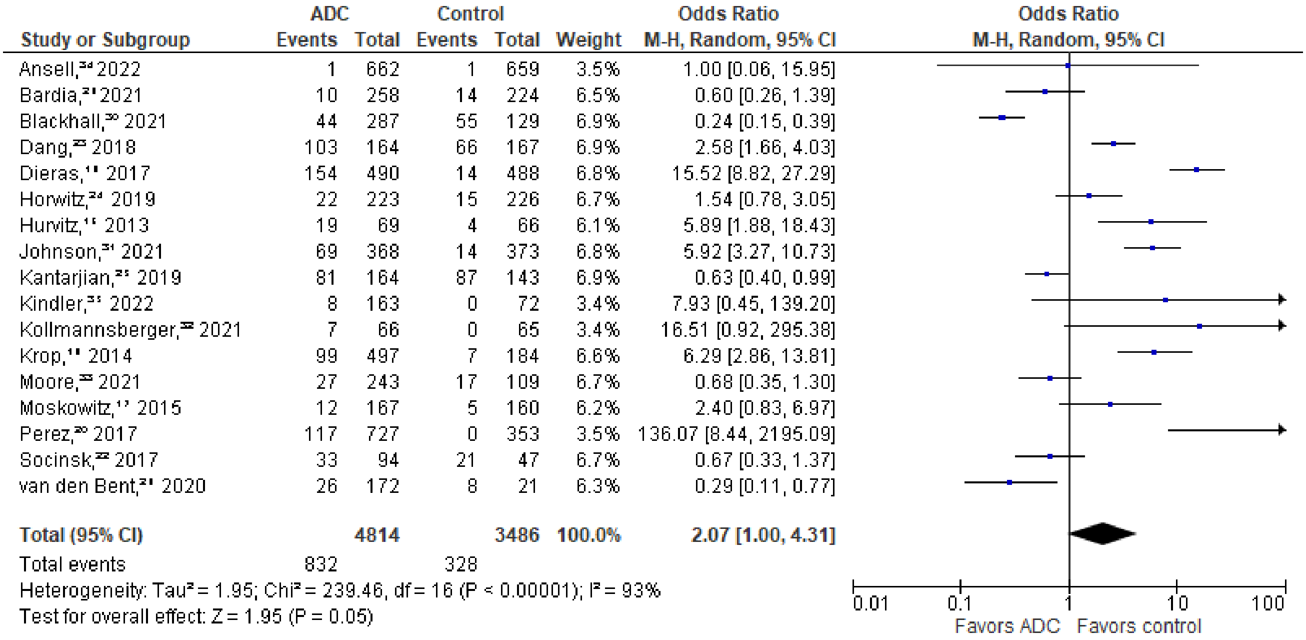

Supplementary Figure 4. Forest plot of incidence of hepatic toxicities

A Alanine aminotransferase increased

Study or Subgroup	ADC Events	ADC Total	Control Events	Control Total	Weight	Odds Ratio M-H, Random, 95% CI
Ansell,[34] 2022	68	662	26	659	8.6%	2.79 [1.75, 4.44]
Bardia,[28] 2021	28	258	22	224	7.7%	1.12 [0.62, 2.02]
Blackhall,[30] 2021	18	287	1	129	1.9%	8.57 [1.13, 64.87]
Dang,[23] 2018	30	164	17	167	7.3%	1.98 [1.04, 3.74]
Dieras,[18] 2017	93	490	48	488	9.2%	2.15 [1.48, 3.12]
Horwitz,[24] 2019	11	223	0	226	1.1%	24.52 [1.44, 418.59]
Hurvitz,[16] 2013	18	69	4	66	4.3%	5.47 [1.74, 17.19]
Johnson,[31] 2021	39	368	8	373	6.4%	5.41 [2.49, 11.74]
Kantarjian,[25] 2019	25	164	18	143	7.2%	1.25 [0.65, 2.40]
Kindler,[35] 2022	20	163	6	72	5.2%	1.54 [0.59, 4.01]
Kollmannsberger,[32] 2021	4	66	3	65	2.9%	1.33 [0.29, 6.21]
Krop,[18] 2014	45	497	10	184	6.8%	1.73 [0.85, 3.51]
Moore,[33] 2021	40	243	6	109	5.6%	3.38 [1.39, 8.24]
Perez,[20] 2017	78	727	10	353	7.1%	4.12 [2.11, 8.07]
Prince,[21] 2017	3	66	5	62	3.1%	0.54 [0.12, 2.37]
Shitara,[27] 2020	9	125	3	62	3.5%	1.53 [0.40, 5.85]
Socinsk,[22] 2017	11	94	0	47	1.0%	13.08 [0.75, 227.04]
van den Bent,[28] 2020	24	172	1	21	1.8%	3.24 [0.42, 25.30]
von Minckwitz,[26] 2019	171	740	41	720	9.3%	4.98 [3.48, 7.12]
**Total (95% CI)**		**5578**		**4170**	**100.0%**	**2.51 [1.84, 3.40]**
Total events	735		229			

Heterogeneity: $Tau^2 = 0.23$; $Chi^2 = 47.95$, df = 18 (P = 0.0002); $I^2 = 62\%$
Test for overall effect: Z = 5.87 (P < 0.00001)

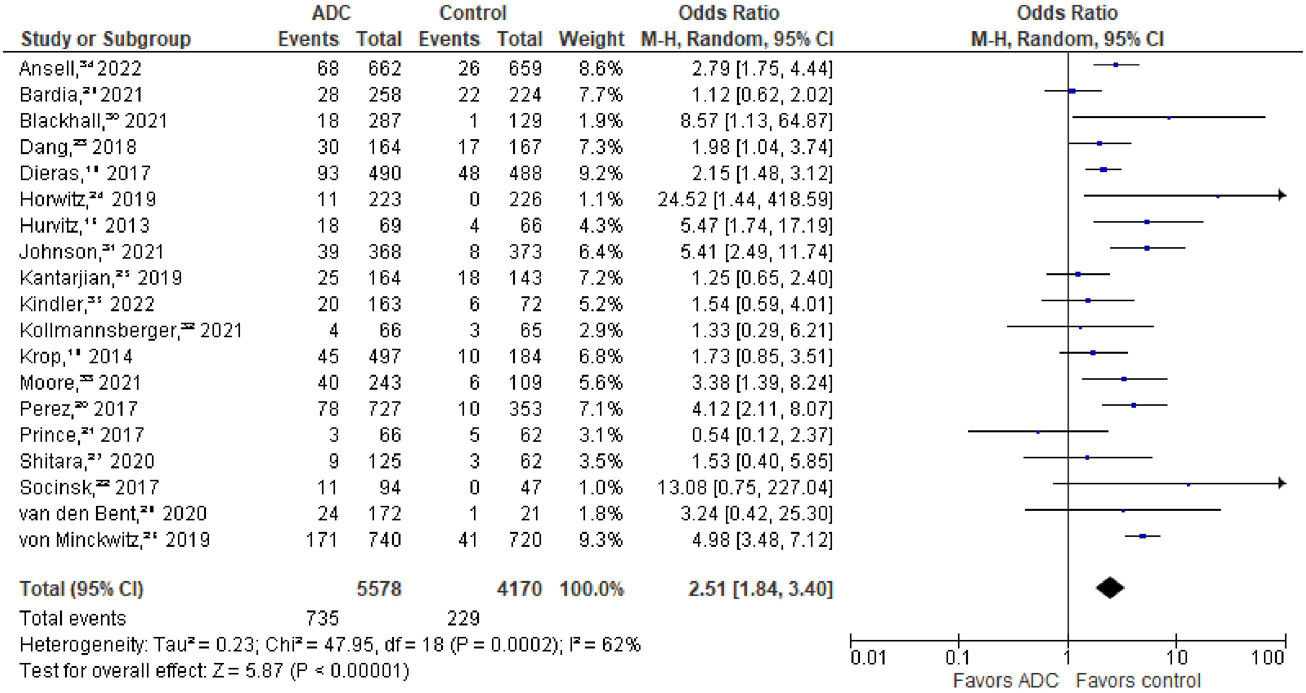

B Aspartate aminotransferase increased

Study or Subgroup	ADC Events	ADC Total	Control Events	Control Total	Weight	Odds Ratio M-H, Random, 95% CI
Ansell,[34] 2022	47	662	19	659	7.6%	2.57 [1.49, 4.44]
Bardia,[28] 2021	30	258	27	224	7.6%	0.96 [0.55, 1.67]
Blackhall,[30] 2021	20	287	3	129	4.1%	3.15 [0.92, 10.78]
Dang,[23] 2018	46	164	18	167	7.3%	3.23 [1.78, 5.86]
Dieras,[18] 2017	123	490	53	488	8.7%	2.75 [1.94, 3.91]
Hurvitz,[16] 2013	30	69	4	66	4.5%	11.92 [3.90, 36.45]
Johnson,[31] 2021	48	368	13	373	7.1%	4.15 [2.21, 7.81]
Kantarjian,[25] 2019	37	164	16	143	7.1%	2.31 [1.22, 4.37]
Kindler,[35] 2022	22	163	4	72	4.6%	2.65 [0.88, 8.00]
Kollmannsberger,[32] 2021	5	66	5	65	3.9%	0.98 [0.27, 3.57]
Krop,[18] 2014	64	497	13	184	7.2%	1.94 [1.04, 3.62]
Moore,[33] 2021	43	243	8	109	6.2%	2.71 [1.23, 5.99]
Perez,[20] 2017	84	727	9	353	6.7%	4.99 [2.48, 10.05]
Prince,[21] 2017	1	66	4	62	1.8%	0.22 [0.02, 2.05]
Shitara,[27] 2020	12	125	3	62	3.8%	2.09 [0.57, 7.69]
Socinsk,[22] 2017	8	94	1	47	1.9%	4.28 [0.52, 35.28]
van den Bent,[28] 2020	22	172	0	21	1.2%	6.43 [0.38, 109.88]
von Minckwitz,[26] 2019	210	740	40	720	8.7%	6.74 [4.72, 9.62]
**Total (95% CI)**		**5355**		**3944**	**100.0%**	**2.83 [2.04, 3.93]**
Total events	852		240			

Heterogeneity: $Tau^2 = 0.29$; $Chi^2 = 57.02$, df = 17 (P < 0.00001); $I^2 = 70\%$
Test for overall effect: Z = 6.23 (P < 0.00001)

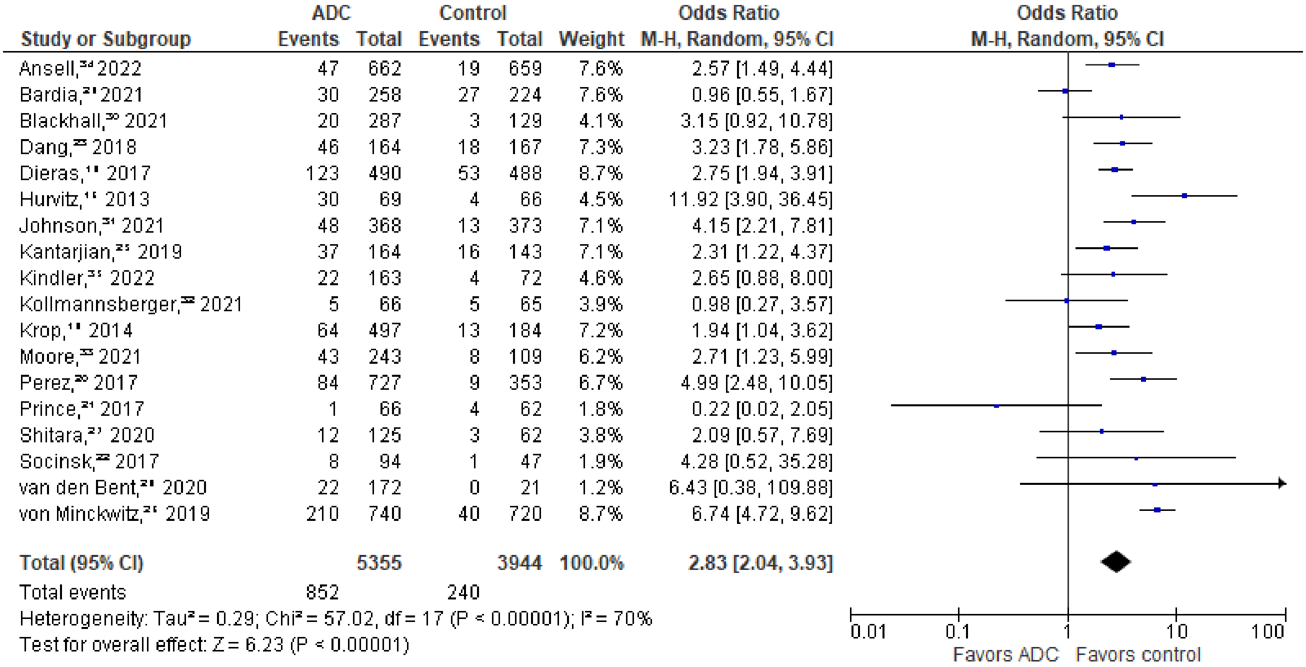

Supplementary Figure 5. Forest plot of incidence of ocular toxicities

A Cataract

Study or Subgroup	ADC Events	ADC Total	Control Events	Control Total	Weight	Odds Ratio M-H, Random, 95% CI
Kindler,[35] 2022	9	163	2	72	37.1%	2.05 [0.43, 9.71]
Moore,[33] 2021	36	243	2	109	43.3%	9.30 [2.20, 39.38]
Shitara,[27] 2020	1	125	0	62	8.7%	1.51 [0.06, 37.50]
van den Bent,[24] 2020	9	172	0	21	10.9%	2.50 [0.14, 44.47]
**Total (95% CI)**		**703**		**264**	**100.0%**	**3.92 [1.52, 10.13]**
Total events	55		4			

Heterogeneity: $Tau^2 = 0.00$; $Chi^2 = 2.62$, df = 3 (P = 0.45); $I^2 = 0\%$
Test for overall effect: Z = 2.82 (P = 0.005)

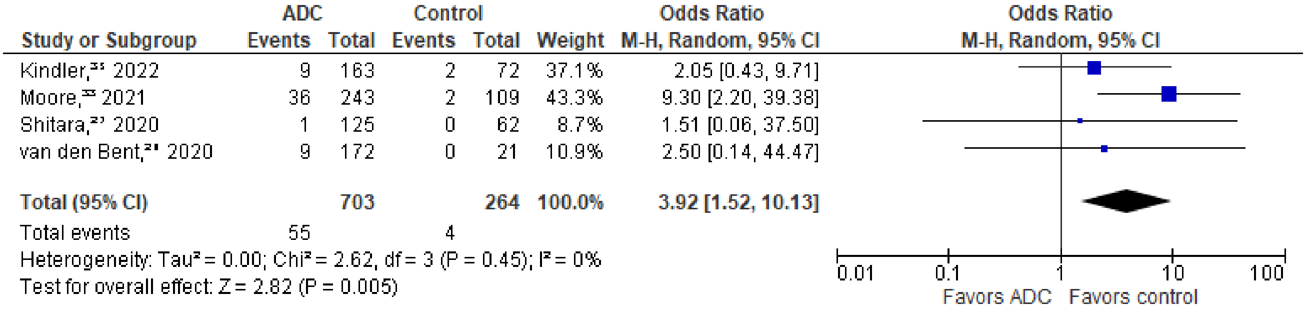

B Eye pain

Study or Subgroup	ADC Events	ADC Total	Control Events	Control Total	Weight	Odds Ratio M-H, Random, 95% CI
Kindler,[35] 2022	3	163	0	72	18.6%	3.16 [0.16, 62.01]
Kollmannsberger,[32] 2021	7	66	0	65	19.8%	16.51 [0.92, 295.38]
Moore,[33] 2021	32	243	1	109	41.1%	16.38 [2.21, 121.49]
van den Bent,[24] 2020	18	172	0	21	20.4%	5.15 [0.30, 88.57]
**Total (95% CI)**		**644**		**267**	**100.0%**	**9.54 [2.64, 34.46]**
Total events	60		1			

Heterogeneity: $Tau^2 = 0.00$; $Chi^2 = 1.16$, df = 3 (P = 0.76); $I^2 = 0\%$
Test for overall effect: Z = 3.44 (P = 0.0006)

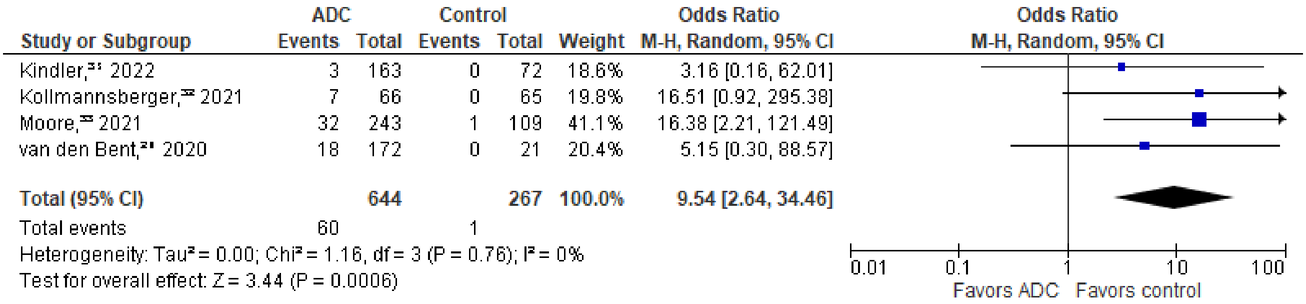

C Photophobia

Study or Subgroup	ADC Events	ADC Total	Control Events	Control Total	Weight	Odds Ratio M-H, Random, 95% CI
Kindler,[35] 2022	1	163	0	72	11.9%	1.34 [0.05, 33.25]
Kollmannsberger,[32] 2021	5	66	0	65	14.4%	11.72 [0.63, 216.33]
Moore,[33] 2021	34	243	2	109	58.6%	8.70 [2.05, 36.92]
van den Bent,[24] 2020	20	172	0	21	15.2%	5.78 [0.34, 99.08]
**Total (95% CI)**		**644**		**267**	**100.0%**	**6.84 [2.26, 20.67]**
Total events	60		2			

Heterogeneity: $Tau^2 = 0.00$; $Chi^2 = 1.26$, df = 3 (P = 0.74); $I^2 = 0\%$
Test for overall effect: Z = 3.41 (P = 0.0007)

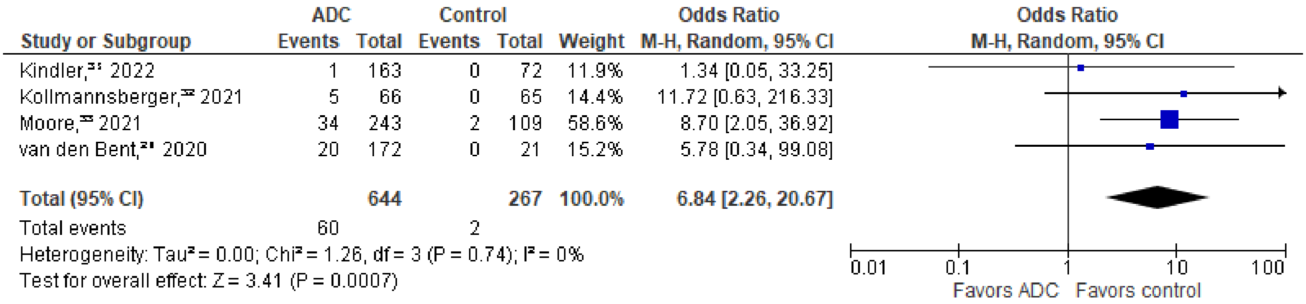

D Vision blurred

Study or Subgroup	ADC Events	ADC Total	Control Events	Control Total	Weight	Odds Ratio M-H, Random, 95% CI
Johnson,[31] 2021	0	368	1	373	8.1%	0.34 [0.01, 8.30]
Kindler,[35] 2022	5	163	0	72	9.2%	5.03 [0.27, 92.21]
Kollmannsberger,[32] 2021	26	66	7	65	21.0%	5.39 [2.13, 13.61]
Krop,[14] 2014	0	497	1	184	8.1%	0.12 [0.00, 3.03]
Moore,[33] 2021	104	243	6	109	21.5%	12.84 [5.43, 30.40]
Perez,[20] 2017	32	727	11	353	22.4%	1.43 [0.71, 2.87]
van den Bent,[24] 2020	47	172	0	21	9.6%	16.27 [0.97, 274.04]
**Total (95% CI)**		**2236**		**1177**	**100.0%**	**3.12 [1.02, 9.55]**
Total events	214		26			

Heterogeneity: $Tau^2 = 1.32$; $Chi^2 = 23.87$, df = 6 (P = 0.0006); $I^2 = 75\%$
Test for overall effect: Z = 2.00 (P = 0.05)

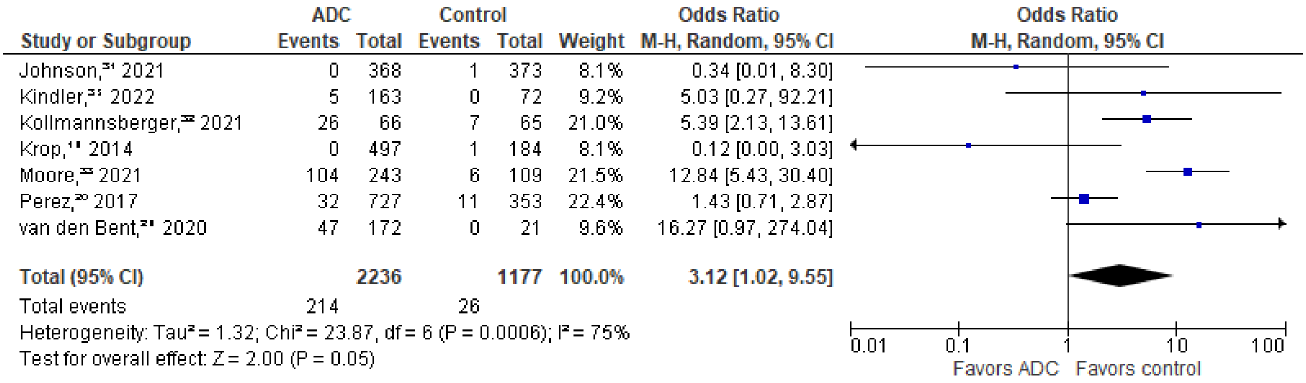

Supplementary Figure 6. Forest plot of incidence of gastrointestinal toxicities

A Abdominal pain

Study or Subgroup	ADC Events	ADC Total	Control Events	Control Total	Weight	Odds Ratio M-H, Random, 95% CI
Ansell,[34] 2022	149	662	69	659	8.1%	2.48 [1.82, 3.38]
Bardia,[21] 2021	57	258	19	224	6.3%	3.06 [1.76, 5.33]
Blackhall,[30] 2021	23	287	12	129	5.1%	0.85 [0.41, 1.76]
Dang,[23] 2018	15	164	14	167	4.9%	1.10 [0.51, 2.36]
Dieras,[18] 2017	47	490	53	488	7.3%	0.87 [0.58, 1.32]
Horwitz,[24] 2019	27	223	23	226	6.1%	1.22 [0.67, 2.19]
Hurvitz,[16] 2013	7	69	4	66	2.7%	1.75 [0.49, 6.28]
Johnson,[31] 2021	27	368	16	373	5.7%	1.77 [0.94, 3.34]
Kantarjian,[25] 2019	22	164	27	143	5.9%	0.67 [0.36, 1.23]
Kindler,[35] 2022	23	163	11	72	4.8%	0.91 [0.42, 1.99]
Kollmannsberger,[32] 2021	15	66	10	65	4.2%	1.62 [0.67, 3.92]
Krop,[18] 2014	41	497	23	184	6.4%	0.63 [0.37, 1.08]
Moore,[33] 2021	82	243	25	109	6.6%	1.71 [1.02, 2.88]
Moskowitz,[17] 2015	21	167	6	160	4.0%	3.69 [1.45, 9.40]
Perez,[20] 2017	77	727	33	353	7.2%	1.15 [0.75, 1.76]
Shitara,[27] 2020	15	125	8	62	4.1%	0.92 [0.37, 2.30]
Socinsk,[22] 2017	5	94	4	47	2.4%	0.60 [0.15, 2.36]
van den Bent,[28] 2020	5	172	0	21	0.7%	1.41 [0.08, 26.43]
von Minckwitz,[26] 2019	58	740	43	720	7.4%	1.34 [0.89, 2.01]
**Total (95% CI)**		**5679**		**4268**	**100.0%**	**1.29 [1.01, 1.66]**
Total events	716		400			

Heterogeneity: $Tau^2 = 0.17$; $Chi^2 = 52.06$, df = 18 (P < 0.0001); $I^2 = 65\%$
Test for overall effect: Z = 2.04 (P = 0.04)

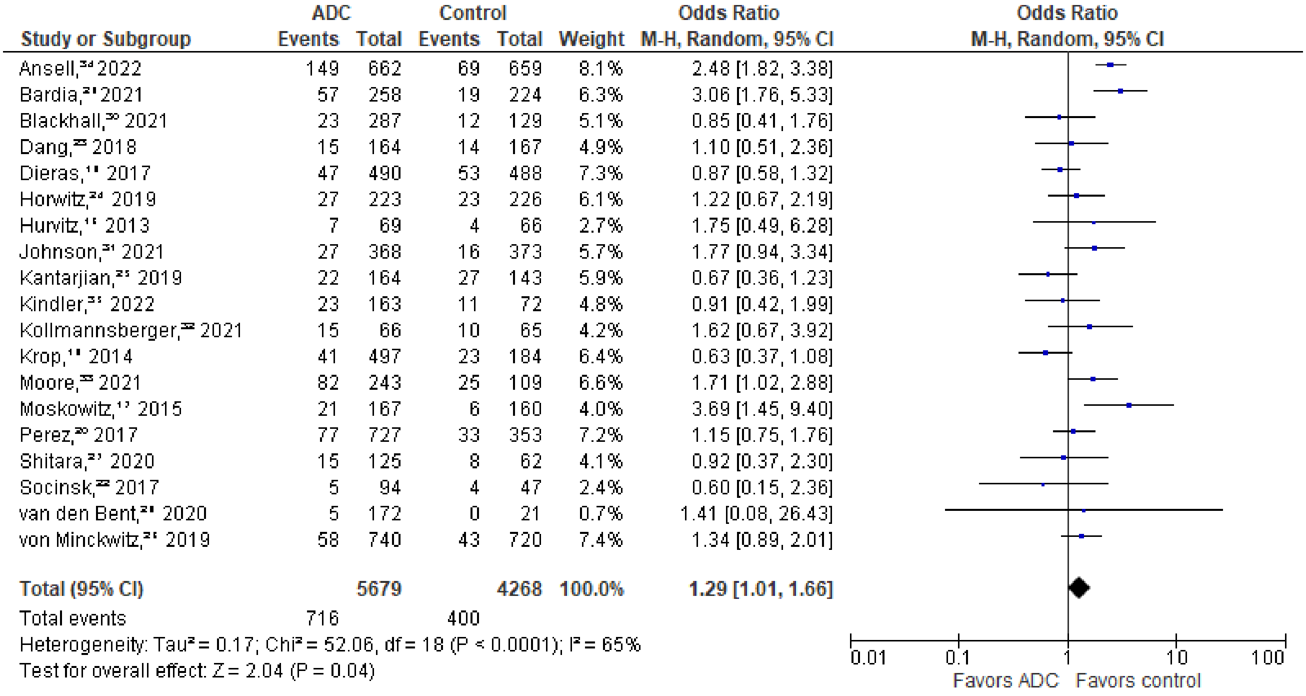

B Abdominal pain upper

Study or Subgroup	ADC Events	ADC Total	Control Events	Control Total	Weight	Odds Ratio M-H, Random, 95% CI
Ansell,[34] 2022	64	662	34	659	20.1%	1.97 [1.28, 3.03]
Bardia,[21] 2021	24	258	8	224	6.4%	2.77 [1.22, 6.30]
Blackhall,[30] 2021	1	287	0	129	0.4%	1.36 [0.05, 33.51]
Dieras,[18] 2017	60	490	45	488	21.8%	1.37 [0.91, 2.07]
Horwitz,[24] 2019	23	223	10	226	7.2%	2.48 [1.15, 5.35]
Hurvitz,[16] 2013	12	69	4	66	3.1%	3.26 [1.00, 10.70]
Johnson,[31] 2021	1	368	0	373	0.4%	3.05 [0.12, 75.09]
Kantarjian,[25] 2019	12	164	12	143	6.2%	0.86 [0.37, 1.98]
Kindler,[35] 2022	9	163	5	72	3.5%	0.78 [0.25, 2.42]
Kollmannsberger,[32] 2021	0	66	1	65	0.4%	0.32 [0.01, 8.08]
Krop,[18] 2014	7	497	1	184	1.0%	2.61 [0.32, 21.39]
Moore,[33] 2021	16	243	10	109	6.3%	0.70 [0.31, 1.59]
Perez,[20] 2017	85	727	31	353	19.9%	1.38 [0.89, 2.12]
Shitara,[27] 2020	5	125	1	62	1.0%	2.54 [0.29, 22.24]
Socinsk,[22] 2017	7	94	2	47	1.7%	1.81 [0.36, 9.08]
van den Bent,[28] 2020	1	172	0	21	0.4%	0.38 [0.01, 9.53]
**Total (95% CI)**		**4608**		**3221**	**100.0%**	**1.53 [1.23, 1.89]**
Total events	327		164			

Heterogeneity: $Tau^2 = 0.01$; $Chi^2 = 15.89$, df = 15 (P = 0.39); $I^2 = 6\%$
Test for overall effect: Z = 3.88 (P = 0.0001)

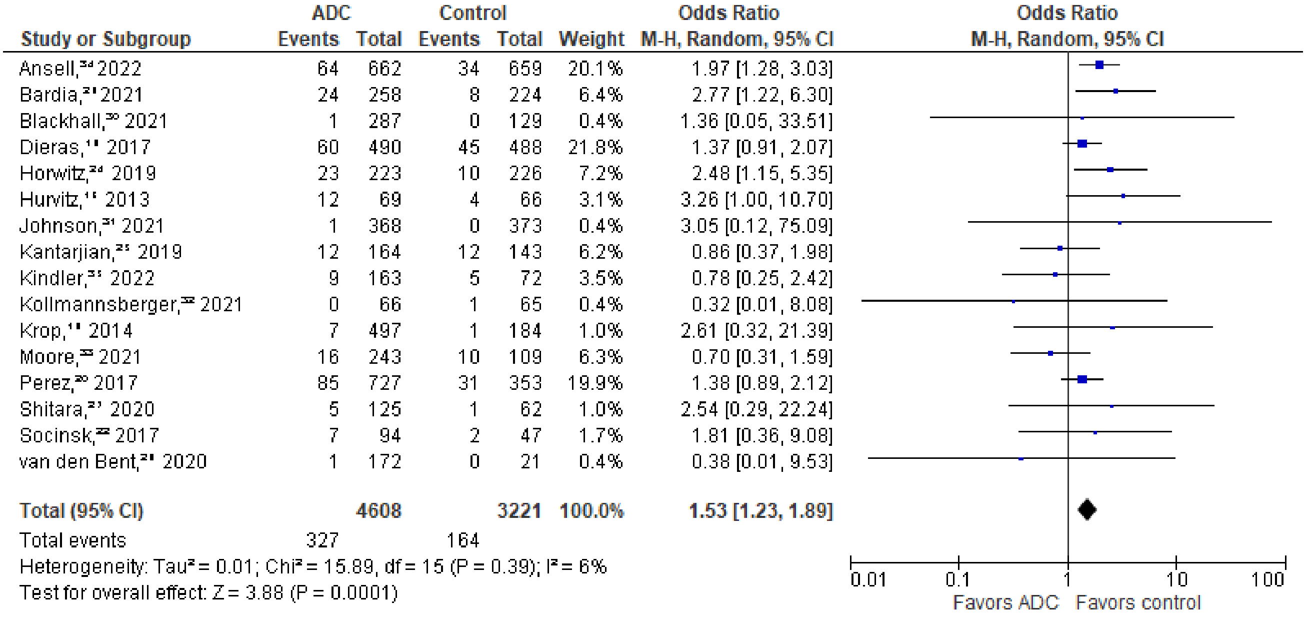

C Dry mouth

Study or Subgroup	ADC Events	ADC Total	Control Events	Control Total	Weight	Odds Ratio M-H, Random, 95% CI
Dieras,[18] 2017	85	490	26	488	20.7%	3.73 [2.36, 5.90]
Hurvitz,[16] 2013	11	69	3	66	10.2%	3.98 [1.06, 14.99]
Kindler,[35] 2022	7	163	0	72	3.2%	6.95 [0.39, 123.32]
Kollmannsberger,[32] 2021	6	66	6	65	11.5%	0.98 [0.30, 3.22]
Krop,[18] 2014	60	497	2	184	9.4%	12.49 [3.02, 51.66]
Moore,[33] 2021	11	243	2	109	8.6%	2.54 [0.55, 11.64]
Perez,[20] 2017	97	727	13	353	18.9%	4.03 [2.22, 7.29]
von Minckwitz,[26] 2019	100	740	9	720	17.6%	12.34 [6.19, 24.62]
**Total (95% CI)**		**2995**		**2057**	**100.0%**	**4.46 [2.56, 7.78]**
Total events	377		61			

Heterogeneity: Tau² = 0.33; Chi² = 18.57, df = 7 (P = 0.010); I² = 62%
Test for overall effect: Z = 5.27 (P < 0.00001)

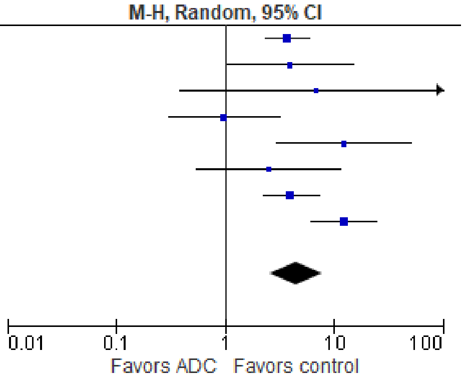

D Gingival bleeding

Study or Subgroup	ADC Events	ADC Total	Control Events	Control Total	Weight	Odds Ratio M-H, Random, 95% CI
Hurvitz,[16] 2013	4	69	0	66	8.2%	9.14 [0.48, 173.12]
Kindler,[35] 2022	1	163	0	72	6.9%	1.34 [0.05, 33.25]
Krop,[18] 2014	21	497	0	184	9.0%	16.65 [1.00, 276.27]
Moore,[33] 2021	2	243	0	109	7.7%	2.27 [0.11, 47.62]
Perez,[20] 2017	56	727	4	353	68.1%	7.28 [2.62, 20.25]
**Total (95% CI)**		**1699**		**784**	**100.0%**	**6.50 [2.80, 15.12]**
Total events	84		4			

Heterogeneity: Tau² = 0.00; Chi² = 1.99, df = 4 (P = 0.74); I² = 0%
Test for overall effect: Z = 4.35 (P < 0.0001)

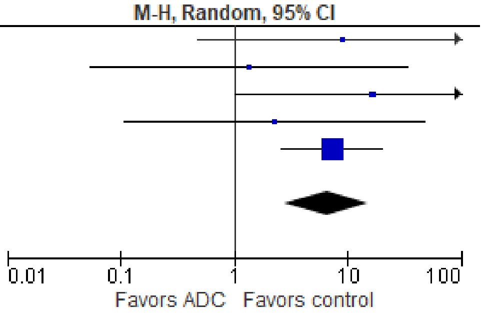

E Oral pain

Study or Subgroup	ADC Events	ADC Total	Control Events	Control Total	Weight	Odds Ratio M-H, Random, 95% CI
Hurvitz,[16] 2013	0	69	4	66	33.4%	0.10 [0.01, 1.89]
Kindler,[35] 2022	0	163	3	72	32.6%	0.06 [0.00, 1.19]
Kollmannsberger,[32] 2021	0	66	5	65	34.0%	0.08 [0.00, 1.53]
**Total (95% CI)**		**298**		**203**	**100.0%**	**0.08 [0.01, 0.44]**
Total events	0		12			

Heterogeneity: Tau² = 0.00; Chi² = 0.06, df = 2 (P = 0.97); I² = 0%
Test for overall effect: Z = 2.92 (P = 0.004)

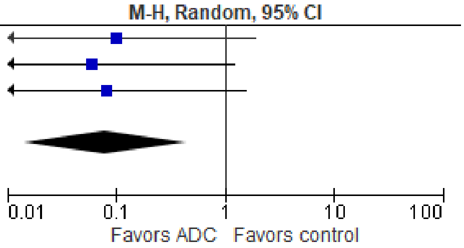

F Toothache

Study or Subgroup	ADC Events	ADC Total	Control Events	Control Total	Weight	Odds Ratio M-H, Random, 95% CI
Hurvitz,[16] 2013	2	69	7	66	63.1%	0.25 [0.05, 1.26]
Kindler,[35] 2022	1	163	1	72	21.1%	0.44 [0.03, 7.11]
Moore,[33] 2021	0	243	1	109	15.9%	0.15 [0.01, 3.68]
**Total (95% CI)**		**475**		**247**	**100.0%**	**0.26 [0.07, 0.93]**
Total events	3		9			

Heterogeneity: Tau² = 0.00; Chi² = 0.25, df = 2 (P = 0.88); I² = 0%
Test for overall effect: Z = 2.06 (P = 0.04)

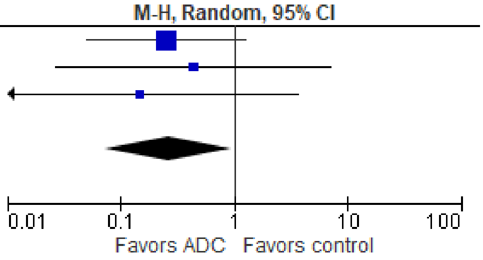

G Vomiting

Study or Subgroup	ADC Events	ADC Total	Control Events	Control Total	Weight	Odds Ratio M-H, Random, 95% CI
Ansell,[34] 2022	223	662	186	659	6.5%	1.29 [1.02, 1.63]
Bardia,[28] 2021	87	258	36	224	5.8%	2.66 [1.71, 4.13]
Blackhall,[30] 2021	31	287	18	129	5.0%	0.75 [0.40, 1.39]
Dang,[23] 2018	23	164	33	167	5.2%	0.66 [0.37, 1.19]
Dieras,[18] 2017	105	490	155	488	6.4%	0.59 [0.44, 0.78]
Horwitz,[24] 2019	61	223	40	226	5.8%	1.75 [1.12, 2.75]
Hurvitz,[15] 2013	18	69	17	66	4.4%	1.02 [0.47, 2.20]
Johnson,[31] 2021	36	368	37	373	5.6%	0.98 [0.61, 1.60]
Kantarjian,[25] 2019	26	164	35	143	5.3%	0.58 [0.33, 1.02]
Kindler,[35] 2022	34	163	5	72	3.6%	3.53 [1.32, 9.45]
Kollmannsberger,[32] 2021	18	66	22	65	4.5%	0.73 [0.35, 1.55]
Krop,[18] 2014	89	497	18	184	5.4%	2.01 [1.18, 3.44]
Moore,[33] 2021	66	243	24	109	5.4%	1.32 [0.77, 2.25]
Moskowitz,[17] 2015	29	167	12	160	4.7%	2.59 [1.27, 5.28]
Perez,[20] 2017	195	727	70	353	6.3%	1.48 [1.09, 2.02]
Prince,[21] 2017	11	66	3	62	2.6%	3.93 [1.04, 14.85]
Shitara,[27] 2020	34	125	5	62	3.6%	4.26 [1.57, 11.53]
Socinsk,[22] 2017	23	94	12	47	4.3%	0.94 [0.42, 2.12]
van den Bent,[28] 2020	24	172	6	21	3.4%	0.41 [0.14, 1.15]
von Minckwitz,[26] 2019	109	740	39	720	6.1%	3.02 [2.06, 4.42]
**Total (95% CI)**		**5745**		**4330**	**100.0%**	**1.32 [1.01, 1.74]**
Total events	1242		773			

Heterogeneity: Tau² = 0.28; Chi² = 104.24, df = 19 (P < 0.00001); I² = 82%

Test for overall effect: Z = 2.03 (P = 0.04)

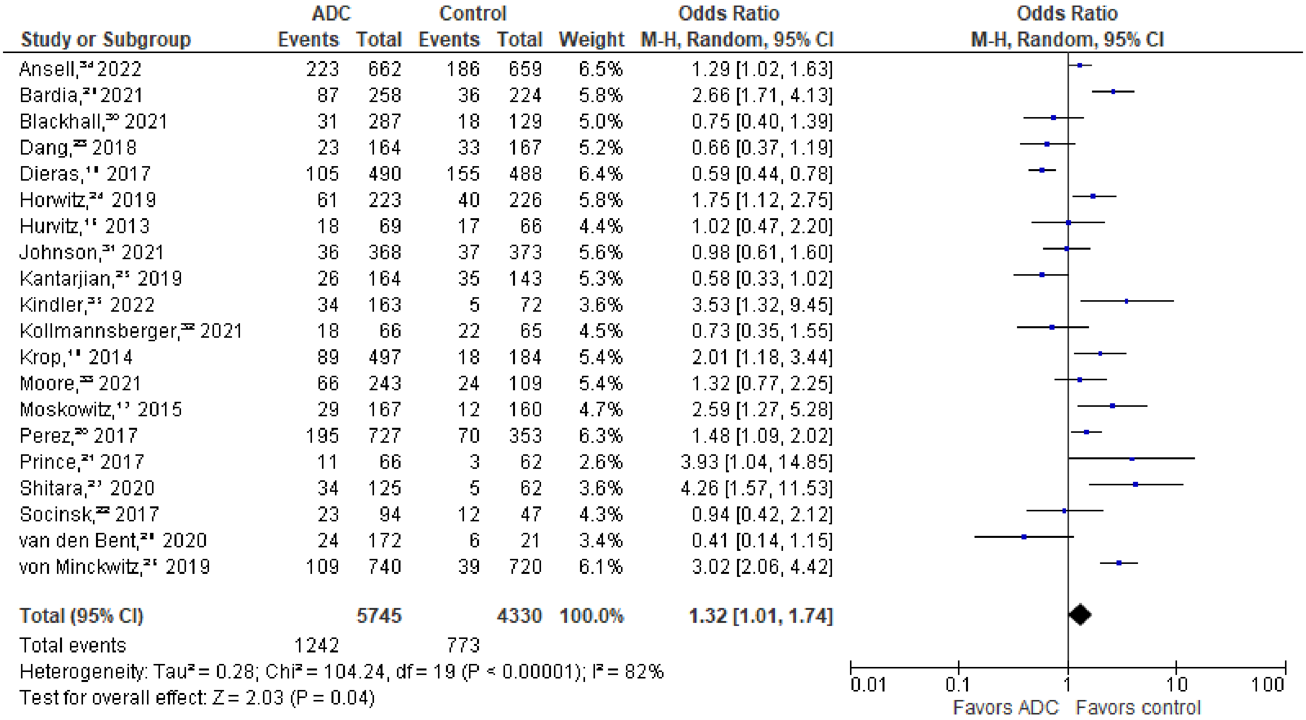

Supplement: pkad069_Supplementary_Data [file pkad069_supplementary_data.pdf]
